# Trace Eyeblink Conditioning in Mice Is Dependent upon the Dorsal Medial Prefrontal Cortex, Cerebellum, and Amygdala: Behavioral Characterization and Functional Circuitry^1^,^2^,^3^

**DOI:** 10.1523/ENEURO.0051-14.2015

**Published:** 2015-07-10

**Authors:** Jennifer J. Siegel, William Taylor, Richard Gray, Brian Kalmbach, Boris V. Zemelman, Niraj S. Desai, Daniel Johnston, Raymond A. Chitwood

**Affiliations:** 1 Center for Learning and Memory, University of Texas at Austin, Austin, Texas 78712; 2 Department of Neuroscience, University of Texas at Austin, Austin, Texas 78712

**Keywords:** anterograde labeling, cerebellum, pontine nuclei, prefrontal cortex, retrograde labeling, trace eyeblink conditioning

## Abstract

Trace eyeblink conditioning is useful for studying the interaction of multiple brain areas in learning and memory. The goal of the current work was to determine whether trace eyeblink conditioning could be established in a mouse model in the absence of elicited startle responses and the brain circuitry that supports this learning. We show here that mice can acquire trace conditioned responses (tCRs) devoid of startle while head-restrained and permitted to freely run on a wheel. Most mice (75%) could learn with a trace interval of 250 ms. Because tCRs were not contaminated with startle-associated components, we were able to document the development and timing of tCRs in mice, as well as their long-term retention (at 7 and 14 d) and flexible expression (extinction and reacquisition). To identify the circuitry involved, we made restricted lesions of the medial prefrontal cortex (mPFC) and found that learning was prevented. Furthermore, inactivation of the cerebellum with muscimol completely abolished tCRs, demonstrating that learned responses were driven by the cerebellum. Finally, inactivation of the mPFC and amygdala in trained animals nearly abolished tCRs. Anatomical data from these critical regions showed that mPFC and amygdala both project to the rostral basilar pons and overlap with eyelid-associated pontocerebellar neurons. The data provide the first report of trace eyeblink conditioning in mice in which tCRs were driven by the cerebellum and required a localized region of mPFC for acquisition. The data further reveal a specific role for the amygdala as providing a conditioned stimulus-associated input to the cerebellum.

## Significance Statement

The current study characterizes the learned behavior and identifies the brain circuitry necessary for trace eyeblink conditioning in mice. We report that under learning conditions designed to avoid elicited startle responses, mice expressed learned responses that were cerebellar-dependent and required a localized region of the dorsal medial prefrontal cortex. Using functional inactivation paired with anatomical tracers, we further identify the trace eyeblink conditioning circuitry in the mouse model that underlies this basic associative learning task, including a more refined role for how amygdala inputs might support this task in the absence of conditioned stimulus-evoked startle.

## Introduction

Awake behaving mice offer many advantages for studying higher cognitive function. However, it can be challenging to develop associative learning and memory tasks in rodents without also engaging startle circuitry, particularly with the use of acoustic stimuli. Rodents show pronounced acoustic startle, which results in a fast motor response (<50 ms) that includes the facial muscles (Koch, 1999). Additionally, startle responses can be either potentiated or habituated with experience, complicating analysis of learned behaviors that result from training. The potentiation of startle is mediated by amygdala activation (Rosen and Davis, 1988), and field potential recordings from the amygdala show excitatory responses in association with the expression of startle (Ebert and Koch, 1997).

One example of an associative learning task that often uses an acoustic stimulus is eyeblink conditioning—a form of associational learning that pairs a neutral conditioning stimulus (CS) with a reflexive unconditioned stimulus (US) to elicit an eyeblink. With repeated pairings, animals learn to close the eyelid in response to the CS and in anticipation of the US (the conditioned response; CR). Two variants of eyeblink conditioning, trace and delay differ in the temporal relationship between the CS and US, and in the underlying brain circuitry. Trace conditioning includes a stimulus-free interval between the CS and US, whereas the stimuli overlap in delay conditioning. Although both trace and delay conditioning require the cerebellum to drive motor output, trace conditioning has been shown to also require forebrain regions, such as the medial prefrontal cortex (mPFC) and hippocampus (Steinmetz et al., 1989; Kim et al., 1995; Kronforst-Collins and Disterhoft, 1998; Weiss et al., 1999; Takehara et al., 2003; Tseng et al., 2004; Kalmbach et al., 2009, 2010b; Siegel and Mauk, 2013; Chen et al., 2014).

In rodents, however, the amygdala may also play an essential role in eyeblink conditioning (Boele et al., 2010). For example, lesions or inactivation of the amygdala resulted in slower acquisition of delay CRs and deficits in asymptotic performance in both rats and mice (Lee and Kim, 2004; Sakamoto and Endo, 2010). Amygdala activation has been shown to facilitate neural responses to stimuli in the basilar pons, a source of forebrain input to the cerebellum, and has been suggested to drive faster acquisition of the CR (Taub and Mintz, 2010). However, many eyeblink studies in rodents that used a tone CS noted evidence of acoustic startle in eyelid responses during at least some trials (Takatsuki et al., 2001; Tseng et al., 2004; Ewers et al., 2006; Brown et al., 2010), and so the putative role of the amygdala may more accurately reflect an interaction between startle and eyeblink circuitry. To further complicate matters, it has also been shown that the amygdala can drive a short latency noncerebellar learned eyelid response (onset at 50–70 ms, peaking at ∼115 ms) that occurs in parallel and overlaps with the well timed cerebellar component (Boele et al., 2010; Sakamoto and Endo, 2010). The two different responses can be dissociated by inactivation of either the cerebellum or amygdala, leaving the residual response intact (Koekkoek et al., 2005; Sakamoto and Endo, 2010). Given that both the mPFC and hippocampus have been shown to interact with the amygdala, it becomes difficult to determine how these brain regions might support trace eyeblink conditioning in rodents when overlapping brain systems are being engaged.

Recently, Medina and colleagues (Chettih et al., 2011; Heiney et al., 2014) reported modified training procedures for delay eyeblink conditioning in mice in which acoustic startle is avoided by using a blue LED as a CS, and mice are permitted to freely run while head-fixed on a wheel allowing for direct and detailed measurement of eyelid movements with high-speed cameras. By implementing those procedures during trace eyeblink conditioning, we show here that mice can acquire trace CRs in the absence of startle and which are completely abolished with cerebellar inactivation. In contrast to previous observations, amygdala inactivation also resulted in CR abolishment, suggesting that under these conditions the amygdala may provide an input to the basilar pons that is part of the CS representation. Using lesions, we identified a restricted region within the mPFC necessary for the acquisition of CRs, and substantiated that a 250 ms trace interval is indeed forebrain-dependent in mice. The data presented here provide the first report of trace CRs in mice whose acquisition is mPFC-dependent and for which the motor response is driven by the cerebellum.

## Materials and Methods

### Subjects and surgical procedures

All surgical and experimental procedures were approved and performed according to the regulations of the authors University’s Institutional Animal Care and Use Committee and were in accordance with the National Institutes of Health guidelines. Male C57BL/6 mice (10–14-weeks-old, The Jackson Laboratory) were shifted to a reverse dark cycle (lights off at 9:00 A.M. and lights on at 9:00 P.M.), at least 2 weeks prior to surgery and remained on this cycle throughout behavioral training. All experiments were performed during the mice’s dark, normally active, period. Both the animal housing facility and the room where training took place were outfitted with red lights in order not to disrupt the reversed light/dark cycle. Training was restricted to between 9:00 A.M. and 7:00 P.M. during the dark cycle, and mice were transported via a covered cart to prevent light exposure. Mice were given food and water *ad libitum* throughout the study.

#### Chronic implant of head restraint bars

All mice were surgically implanted with a custom-fabricated titanium “headplate” for head fixation on a running wheel during training. In addition to different groups of mice used for standard training (trace 50–250, *n* = 16) or training to different intervals (trace 50–350, *n* = 8; trace 50–450, *n* = 8), different naïve groups of mice were also subjected to aspiration lesions or sham surgeries (*n* = 32), injections of anatomical tracer and/or were implanted with a guide cannula for infusions (cerebellum, *n* = 8; amygdala, *n* = 8; see below). In preparation for surgical procedures, mice were initially anesthetized with 3% isoflurane mixed with medical grade oxygen and maintained at 1–2% isoflurane throughout procedures. Animals were then placed in a stereotaxic apparatus and injected with 0.15 ml of Rimadyl (1mg/ml, s.c.). Mice receiving lesions, injections, or cannula implants also received 0.03 ml of dexamethasone (2 mg/ml, s.c.). The skull was prepared by scalping the crown and removing the fascia, and then was scored with the tip of a scalpel blade. After the skull was cleaned and dried, a layer of low viscosity cyanoacrylate was applied over the surface of the exposed skull. An initial layer of Metabond (Parkell) was applied over the cyanoacrylate, the titanium headplate placed, and additional Metabond used to cement the headplate to the skull. Mice were given a minimum of 1 week to recover before beginning acclimation to the running wheel and behavioral training (mice receiving lesions of the mPFC were given 2 weeks to recover, see below).

#### PFC lesion surgeries

Sixteen naïve mice received bilateral aspiration lesions of the mPFC (anterior cingulate, ACc, and medial agranular regions, AGm). After preparing the skull as described above, eight animals received a rectangular craniotomy over the midline (while keeping the sagittal sinus intact) targeted between 0.5–1.5 mm anterior to bregma and extending laterally 1.5 mm on each side. The eight remaining animals received the same craniotomy but targeted between 1.5–2.5 mm anterior to bregma. Tissue was aspirated using a blunt needle attached to a vacuum flask. The region was aspirated to a depth of 1 mm at the lateral half of the craniotomy and 1.5 mm at the midline half. The craniotomy was filled with Kwiksil (World Precision Instruments) and a headplate was implanted as described above. Sixteen control mice were given the same craniotomies, which were also protected with Kwiksil, but no tissue was aspirated. Lesioned and sham control mice were given 2 weeks to recover before acclimation to the running wheel.

#### Virus injection and chronic infusion cannula implants

Eight naïve mice were injected with a recombinant adeno-associated virus (rAAV) into the mPFC to express the tdTomato fluorescent protein from a pan-neuronal synapsin promoter. A guide cannula was also implanted in the cerebellum to allow for *in vivo* infusions (see below). First, the mice and skull were prepared as described above. Animals received a craniotomy over the caudal mPFC at a coordinate for which learning was prevented in lesioned mice (0.85 mm anterior to bregma, 0.5 lateral, contralateral to the trained eye). rAAV (30 nl) was pressure injected (Nanoject, Drummond Scientific) at three locations along the dorsal-ventral axis (1.2, 1.1, and 1.0 mm ventral to the surface of the brain). The injection pipette was left in place for 1–2 min before it was extracted from the brain and the craniotomy sealed with Kwiksil. A second craniotomy was made over the anterior interpositus nucleus of the cerebellum (1.6 mm posterior to lambda, 2.1 mm lateral, ipsilateral to the trained eye). A guide cannula was implanted with the tip lowered 1.0 mm below the brain surface (Plastics One, cut to length 3 mm below a 5 mm pedestal) and secured with cyanoacrylate gel. The headplate was then implanted as described above. Separate groups of naïve mice were also implanted with the same kind of guide cannula either on the brain surface above the mPFC (*n* = 8, 1.25 mm anterior to bregma and 0.5 mm lateral, dura was removed) or bilaterally over the central nuclei of the amygdala (*n* = 8, 1.46 mm posterior to bregma, 3.1 mm lateral, lowered 3.0 mm below bregma), and prepared with a headplate as described above. One mouse from the amygdala group had to be euthanized early in training due to an unrelated foot injury.

### Standard behavioral training

**Figure 1:**
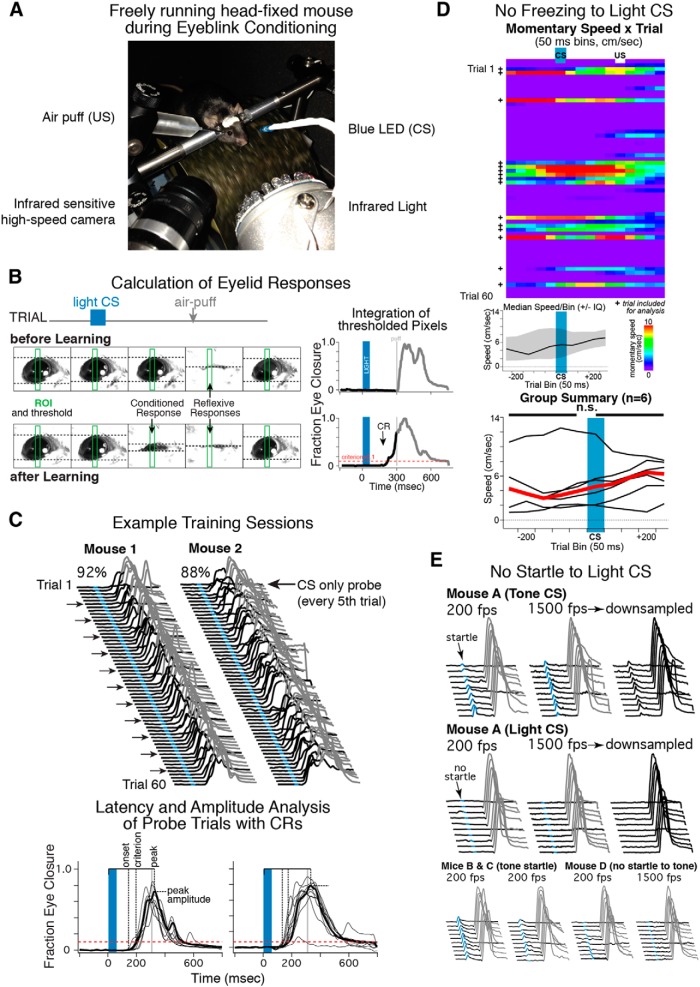
Apparatus and equipment configuration used for trace eyeblink conditioning in head-fixed mice (***A***), analysis of high-speed video (***B***), and example behavioral sessions from two mice (***C***). Mice did not show freezing (***D***) or startle responses (***E***) to presentation of a blue light CS. *A*, Mice were surgically implanted with a headplate to allow for fixation on a running wheel during training. CS included a blue LED CS and an air puff US. Eyelid behavior was monitored during trials with an infrared sensitive high-speed camera (200–250 fps). The black fur around the eye was illuminated with infrared light to contrast with a mouse’s eye and allow for analysis (see below). ***B*,** Standard trace conditioning trials consisted of a 200 ms baseline, 50 ms blue light CS, and a 250 ms stimulus-free trace interval followed by a 20 ms air puff US (top). Each frame of a trial was analyzed by calculating the ratio of white–black pixels within a specified region of interest (green rectangle in example sample frames; dashed lines show borders of white and black thresholded pixels), yielding the FEC at each sampled time point during a trial (right graphs; upward deflection indicates closure, red dashed line shows the minimum FEC to qualify as a CR, 0.1). Initially the mouse closed his eye only in response to the air puff (top), but with continued training learned to close his eye in response to the light CS and in anticipation of the air puff (bottom, CR). ***C***, Waterfall plots show each trial of a session from two well trained mice (blue indicates presentation of light CS, gray indicates presentation of air puff US). Training sessions consisted of 60 trials, with CS-only probe trials presented every fifth trial (arrows) to allow for analysis of CRs in the absence of reflexive US responses. Note the upward deflection of the black lines prior to the air puff presentation for many of the trials, indicative of a CR. Bottom graphs show probe trials from the example sessions, which were used to calculate the latencies and amplitude of CRs for each mouse/session (see text). Note the absence of apparent startle responses just after CS presentation and the ramping topography of eyelid closures such that the peaks of CRs were well timed to US presentation (which were not presented during probe trials). ***D***, Example wheel-running behavior from one mouse during its first trace eyeblink conditioning session (pseudocolor plot, each row is one trial, “+” indicates trials in which mouse was running before CS onset and included in analysis), and the averaged momentary speed before and after CS presentation (middle graph, median ± IQR). Bottom graph shows the averaged momentary speed for six mice (black lines), indicating that mice did not decrease locomotion (freeze) in response to the light CS (paired *t* = −1.31, df = 5, *p* = 0.25, n.s.). **E.** Example eyelid responses from four mice subjected to a startle-eliciting tone (top and bottom rows) or blue light CS (middle row), sampling at either 200 or 1500 fps as indicated. Startle responses to the tone were clearly detected when sampling at 200 fps, and were never observed in response to the light CS, even when sampling at 1500 fps.

Head-fixed mice were trained in custom-built sound-attenuating chambers. Animals were positioned on a cylindrical wheel that allowed them to run freely, according to the methods of Medina and colleagues (Fig. 1*A*; Chettih et al., 2011; Heiney et al., 2014). Training chambers contained an infrared light source for video illumination and a high-speed infrared camera (Prosilica GC 650 or GE 680, Allied Vision Technologies) to capture eyelid position at 200 or 250 fps, respectively, which was counterbalanced within training groups. Higher sampling rates than the maximum full-frame rate possible for each camera were achieved by specifying a subframe that surrounded the eye from the full frame and only sampling from that subframe during trials (Fig. 1*B*). Care was taken to ensure that the infrared light used to illuminate the fur surrounding the eye could not induce photothermal retinal damage by using a dispersed infrared LED array (FY-4748, Shenzhen Feyond Technology). The CS was delivered with a blue LED (HLMP-AB74-WXBDD, Avago Technologies; current = 6.2 mA at 2.78 V) mounted in view of both eyes 3–5 cm in front of and above the head. The air puff US was delivered using a 24 gauge stainless steel cannula positioned 2–4 mm lateral to the right eye (Fig. 1*A*). Each training chamber also included a loudspeaker to provide white noise during training (65 dB at the site of head fixation).

#### Acclimation

After recovery from surgery, mice were acclimated to the training environment and handling procedures in three steps over 5 d before behavioral training began. On day 1, mice were lightly anesthetized with isoflurane and placed in a clean cage (mice typically recovered within 5–10 s of placement). Mice recovered with the experimenter’s hand in close proximity to acclimate mice to the procedures used to place animals on the head-restrained freely moving wheel. On day 2, mice were again lightly anesthetized, but then placed on the wheel to recover. Mice were given 15 min to acclimate to the wheel and training box. On days 3–5, mice were placed on the wheel using the same procedures, and the eyeblink conditioning software was run using the standard parameters used for training (see below) except that no stimuli were presented.

#### Standard training protocol

Training sessions were controlled by custom software (Igor Pro, Wavemetrics, and OpenCV, R. Gray) and consisted of 12 blocks of five trials for a total of 60 trials per session. The intensity of the air puff was adjusted for each mouse before a session and set to the minimum required to elicit a full eyelid closure. The first trial of every block began with a CS-only probe trial, with the next four trials pairing the US and CS (Fig. 1*C*). The time interval between trials was randomized to an average of 15 ± 5 seconds. Standard training trials consisted of a 200 ms baseline and began with a 50 ms blue light CS (LED, 470 nm) followed by a 250 ms stimulus-free interval and terminating with 20 ms eye puff US (Trace 50–250; Fig. 1*B*, top). Different groups of mice were trained with trace intervals of 350 ms (Trace 50–350; *n* = 8) or 450 ms (Trace 50–450; *n* = 8) to investigate differences in the timing of CRs. An additional group of naïve mice (*n* = 8) received pseudoconditioning to test whether mice develop eyelid closures with repeated light presentations purely as a result of sensitization. To maintain a comparable density of CS presentations for these sessions relative to a typical training session, mice were presented with the 50 ms CS on four of every five trials, receiving only the eye puff US on every fifth trial. All other procedures were the same for these sessions.

#### Evaluation of frame rate and likelihood of detecting startle in eyelid responses

Four mice used for pseudoconditioning were later subjected to presentations of the light CS (20 trials) followed by trials in which an acoustic stimulus intended to evoke startle was presented (20 trials, 2 kHz pure tone with a steep onset at 95 dB as measured at the mice’s nearest ear; Koch, 1999). This was performed in a different location with a separate set of equipment to avoid any potential contamination of the main training environment. To achieve the fastest possible frame rate the camera was rotated 90° and the subframe was limited to just larger than the rectangle (ROI) used for analysis (see Analysis). This allowed for sampling rates in excess of 1500 fps. For each of the four mice, 10 trials of light CS sampled at 200 fps were recorded, followed by 10 trials of light CS sampled at 1500 fps. The same was then repeated for trials in which the light CS was replaced with the 50 ms startling tone. Eyelid responses (FECs; see Analysis) generated from 1500 fps were downsampled to 187 fps to determine whether essential information was lost at the lower frame rate (Fig. 1*E*).

#### Long-term memory test and extinction/reacquisition training

Ten of the 12 mice that learned the trace 50–250 protocol were given a 7 d break to test retention of the task (1 mouse did not reach learning criterion until the last standard training session and 1 mouse developed context-associated teariness and so were excluded from the memory test). During the 7 d break, mice were housed as usual but were not removed from the animal facility. The behavior of one mouse did not recover well from the break after four additional training sessions and was excluded from extinction training. Therefore, nine mice proceeded to be tested for extinction and reacquisition. For extinction training, mice were given unpaired CS-only probe trials for the entire 60-trial session daily for 3 d. Mice then experienced reacquisition training, during which mice again received paired trials (12 blocks of 5 trials, consisting of 1 CS-only probe trial and 4 CS-US paired trials per block). Mice received 4 d of reacquisition sessions.

#### Medial PFC lesion training procedures

Aspiration lesions were used to test the necessity of the mPFC during the acquisition of trace CRs, given that pharmacological or viral inactivation include potential uncertainties in the day-to-day efficacy of functional disruption inherent in those manipulations. Naïve mice receiving mPFC lesions (*n* = 16) and the corresponding controls (*n* = 16) were trained using the trace 50–250 protocol with the same standard procedures described above. Lesioned mice and corresponding controls were trained in two groups, the first receiving more caudal mPFC lesions (or craniotomies for controls animals) and the second group receiving more rostral lesions. Four of the control mice from the first group that learned went on to receive a 14 d break to test for long-term memory of the task. If a lesioned mouse did not learn the trace conditioning task, they were then trained to a non-forebrain-dependent delay conditioning protocol. This was necessary to determine whether the learning deficit was specific to trace conditioning or due to a global learning impairment. For delay training, lesioned nonlearners were reassigned to different training boxes after experiencing a 2 week break. Preliminary data suggested that intact mice that did not learn trace conditioning also failed to learn delay conditioning if immediately switched to the new training protocol with no changes in context (such as a “different” box, data not shown). A delay 150 training protocol was used because mice typically learn this interval within 10 training sessions (Heiney et al., 2014). For delay trials, the CS light was presented for 170 ms, and coterminated with a 20 ms air puff US (150 ms interstimulus interval). These animals received the same number of CS only probe trials and number of blocks per session for delay conditioning as was used for trace conditioning.

#### Cerebellar infusion procedure

To reversibly inactivate the eyeblink region(s) of the cerebellum (anterior interpositus nucleus and/or eyeblink region of the cerebellar cortex), 100 nl of 1 mm muscimol (Tocris) dissolved in artificial CSF [aCSF; containing the following (in mm): 125 NaCl, 2.5 KCl, 1.25 NaH_2_PO_4_, 25 NaHCO_3_, 2 CaCl_2_, 2 MgCl_2_, and 12.5 dextrose; pH adjusted to 7.4 after addition of muscimol] was infused before the training session. The solution was loaded into a Hamilton syringe fitted with Tygon tubing and a sterile infusion cannula (Plastics One, 33 gauge cannula that projected 1.8 mm from the bottom of the guide) and manually pressure injected at a rate of 25 nl every 15 sec. The training session began 15 min after the infusion to allow the muscimol to diffuse. Mice were given 2–3 d of additional training sessions before receiving a control infusion of a 10% solution of AlexaFluor-conjugated dextran amines (488, 3000 MW, Invitrogen) dissolved in aCSF. The same procedures were used for control and muscimol infusions. Mice received a final training session on the day following control infusions.

#### Prefrontal cortex and amygdala infusion procedures

The same procedures used to inactivate cerebellar regions were used for unilateral reversible inactivation of the mPFC or bilateral reversible inactivation of amygdala central nuclei (100 nl of 1mm muscimol manually pressure injected at a rate of 25 nl every 15 s, for each hemisphere in cases of amygdala inactivation). After 2–3 d of postmuscimol training sessions, these mice also received control infusions of a 10% solution of AlexaFluor-conjugated dextran amines dissolved in aCSF (AlexaFluor 488–10,000 MW in the side ipsilateral to the trained eye, and AlexaFluor 594–10,000 MW contralateral to the trained eye; Invitrogen).

### Analysis

Off-line analysis of the individual video frames captured for each trial of a session was performed using custom software (Igor Pro, Wavemetrics). A rectangular region-of-interest (ROI; approximately 30 × 200 pixels for the Prosilica GE680 camera and 20 × 130 pixels for the Prosilica GC650 camera) was drawn over a central portion of the eye and applied to each frame (Fig. 1*B*). Pixel values were thresholded such that the binary image reflected the delineation between the eye (viewed as black pixels) and the surrounding fur (represented as white pixels). When the eye is fully open the ratio of black to white pixels within the ROI is low, whereas this ratio increases as the eyelids close [reported as fraction of eye closure (FEC); Fig. 1*B*; Chettih et al., 2011; Heiney et al., 2014]. The observed FEC for each frame of a trial was plotted and reflects the momentary position of the eyelids during a given trial.

#### CR and general performance criteria

Trial-associated FEC plots were used to quantify eyelid behavior over training sessions (Fig. 1*B*,*C*). The mean baseline FEC was calculated for the 200 ms preceding the CS, and this value was subtracted from each sampled point in the trial such that a value of zero represented a maximally open state. Each trial was low-pass filtered using a least squares polynomial-smoothing algorithm (Savitzky–Golay, 2^nd^ order, *n* = 13; Savitzky and Golay, 1964). Trials with baseline eyelid movements that exceeded a SD = 0.015 were excluded from analysis for that session. A CR was defined as an FEC > 0.10 in the period between CS and US onsets (Fig. 1*B*,*C*). Session performance was defined as the number of trials for which a CR was detected divided by the number of included trials for that session. Learning curves and performance before and after manipulations were generated by plotting a mouse’s session performance (CR rate as a percentage) against consecutive training days.

#### CR latencies and amplitude

Various timing and amplitude components of CRs were analyzed to investigate potential changes in behavior during learning or as a result of manipulations. Because reflexive responses to the US can interrupt the topography of learned responses (Fig. 1*B*, bottom right graph), quantification of latencies and amplitude was restricted to CS-only probe trials. Amplitude was defined as the maximum FEC observed between CS onset and the end of the trial (Fig. 1*C*, bottom). The latency to peak was defined as the time between CS onset and peak amplitude (Fig. 1*C*, bottom). Two other measures of timing were also quantified. The latency to criterion was defined as the time between CS onset and CR criterion (when the FEC was ≥0.10; Fig. 1*C*, bottom). The latency to onset was defined as the time between CS onset and the time at which the slope of the FEC response first exceeded zero (Fig. 1*C*, bottom). For each trial with a CR, the slope between the FEC at the point at which criterion was met and three data points before that was calculated. This was performed iteratively for each preceding pair of points until 10 consecutive data points had a slope that approached “0” (if the slope was <0.0001). The first data point that met this condition was considered the initial departure from baseline and was taken as the latency to CR onset for that trial. Statistical comparisons were made for amplitude and/or latency measures between manipulations and between groups of mice trained at different trace intervals by taking the median measure observed for each mouse for a given session and comparing between groups of mice as paired or unpaired data (Student’s *t* test, or Wilcoxon–Mann–Whitney two-sample rank test or the Wilcoxon signed rank test in cases when a given sample size <6, with Bonferroni corrections to control for familywise error, α = 0.05; Table 1). It should be noted that for sample sizes <6 only large effect sizes will be detected, whereas smaller but reliable differences will appear nonsignificant.

**Table 1:** Identification of statistical tests used and parameters relevant to *post hoc* power analysis

	Data	Test	Power (%)	Effect size	Noncentrality	*n*
a'	Unknown	Paired *t* test	9	0.28	0.70	6
b'	Unknown	Paired *t* test	5	0.01	0.02	8
a	Normal	Paired *t* test	70	0.79	2.73	12
b	Unknown	Paired *t* test	39	0.53	1.85	12
c	Normal	1 sample *t* test	6	0.07	0.25	12
d	Unknown	Wilcoxon rank	91	2.11	3.57	4,12
e	Unknown	Wilcoxon signed rank	5	0.11	0.21	4
f	Unknown	Wilcoxon rank	9	0.39	0.65	4,12
G	Unknown	Wilcoxon rank	81	1.81	3.07	4,12
H	Unknown	Wilcoxon rank	6	0.27	0.35	3,4
I	Unknown	Wilcoxon rank	9	0.58	0.74	3,4
J	Unknown	Wilcoxon rank	5	0.006	0.007	3,4
K	Normal	1 sample *t* test	100	1.73	6.47	14
L	Unknown	Mann–Whitney–Wilcoxon	8	0.36	0.59	4,10
M	Unknown	Paired *t* test	100	1.52	5.56	14
N	Unknown	Paired *t* test	15	0.27	1.0	14
P	Normal	Paired *t* test	97	1.11	4.14	14
Q	Unknown	Paired *t* test	6	0.07	0.26	14
R	Unknown	Paired *t* test	10	0.19	0.71	14
S	Normal	Paired *t* test	80	1.07	3.21	9
T	Unknown	Paired *t* test	5	0.03	0.10	9
U	Unknown	Paired *t* test	5	0.06	0.17	8
V	Unknown	Paired *t* test	5	0.06	0.18	8
W	Unknown	Paired *t* test	1	4.86	10.86	5
X	Unknown	Paired *t* test	48	1.13	2.52	5
Y	Unknown	Paired *t* test	100	11.48	28.11	6
Z	Unknown	Paired *t* test	14	0.43	1.06	6
y'	Unknown	Paired *t* test	100	5.82	14.27	6
z'	Unknown	Paired *t* test	47	0.96	2.34	6

Data structure was tested for normality when parametric tests were applied using Kolmogorov–Smirnoff tests, but was of limited application here due to the modest sample sizes inherent in behavioral studies (<20 animals/group). In all cases, nonparametric tests yielded the same results. Effect size and measures of predicted noncentrality are also given to properly infer cases in which *post hoc* calculations of statistical power were weak. In most cases of weak power the effect sizes were medium to large (>0.35). In cases for which the effect size was very small (<0.10) the predicted noncentrality measures were also very low (<0.30), indicating that any potentially real differences between groups was minimal such that additional sampling within reasonable limits would be unlikely to result in statistical significance. It should be noted that only substantial changes in behavior were of interest in the current work. *Post hoc* power analysis was performed using G*Power (v3.1.9.2, www.gpower.hhu.de).

#### Analysis of wheel-running behavior for CS-associated freezing

Wheel-running behavior during training sessions was recorded from mice implanted with bilateral guide cannula for amygdala inactivation (*n* = 6 learners). For this purpose, each wheel was fitted with concentric black-and-white quadrature patterns glued to the side. In the quadrature pattern, black–white repeating squares form a pair of rings around the wheel perimeter that are 90° out of phase with each other. Wheel movement was recorded by measuring the infrared light reflected off each ring with an LED paired with a phototransistor (Sparkfun). The number of black–white transitions were detected for sampling epoch (50 ms) and used to calculate momentary speed (converted to cm/s) by an Arduino microcontroller (www.arduino.cc), which was written to a file along with a timestamp. To test whether mice were freezing to presentation of the light CS during the first acquisition session, analysis was first restricted to trials in which mice were moving the wheel (>0 cm/s) for the five consecutive samples prior to CS presentation (250 ms). The number of trials meeting this movement criterion ranged between 12–40% (median = 31%) for a given session. For these trials, the median speed was calculated for each of the five samples prior to CS presentation (pre-CS bins) and for the five samples including and following CS presentation (post-CS bins; Fig. 1*D*). The average of the pre-CS bins and the average of the post-CS bins was calculated for each mouse and compared with a paired *t* test (α = 0.05).

### Histological procedures

Mice were given lethal intraperitoneal injections of 0.15 ml ketamine mixed with xylazine (10 mg/ml xylazine in 90 mg/ml ketamine) and then perfused intracardially with oxygenated, cold (∼4°C) modified aCSF (2.5 mm KCl, 1.25 mm NaH_2_PO_4_, 25 mm NaHCO_3_, 0.5 mm CaCl_2_, 7 mm MgCl_2_, 7 mm dextrose, 205.5 mm sucrose, 1.3 mm ascorbic acid, and 3.7 mm pyruvate) followed by 4% paraformaldehyde in 0.02 m phosphate buffer. Brains were cryoprotected in a 30% sucrose/4% paraformaldehyde solution overnight or until equilibrated. Tissue was sectioned at 50 µm using a sliding microtome (Leica Microsystems) equipped with a temperature controlled freezing stage (Physitemp). Coronal sections containing the mPFC, amygdala, pons, or cerebellum were taken and transferred to 0.9% physiological saline and mounted on Microfrost Plus slides (Fisher Scientific). Mounted sections were protected from light and dust, and air-dried overnight. Prior to coverslipping, sections were washed in 50% EtOH (1 min), 100% EtOH (3 min), cleared in xylenes for 6–10 min and coverslipped with DPX mounting medium (Electron Microscopy Services).

#### Fluorescence microscopy

Images of brain sections were acquired as described previously (Moya et al., 2014). Structural damage from mPFC lesions was imaged using blue wavelength excitation light to visualize tissue autofluorescence. For animals injected with tdTomato in the mPFC and infused with AlexaFluor 488-conjugated dextran in the cerebellum, the red wavelength excitation light was used to image the tdTomato, green wavelength excitation to image the AlexaFluor 488, and blue wavelength excitation light to visualize structural information via autofluorescence. Overlap of mPFC synaptic terminals and retrogradely labeled cell bodies in the pons was determined using image projections from combined in-focus pixels from a stack of images containing the entire *z*-axis. Following alignment of tiled images and assembly into a single continuous image using the AxioVision software, individual channels were exported as unmodified 8- or 12-bit TIF files. An identical procedure was used to image AlexaFluor 488 or AlexaFluor 594 labeled amygdala terminals in the pons.

Images were processed using ImageJ (NIH; http://rsbweb.nih.gov/ij) or FIJI (Schindelin et al., 2012). Images were adjusted to maximize the distribution of pixel values in an effort to increase contrast and minimize background autofluorescence as appropriate.

#### mPFC lesion and cerebellar or amygdala infusion reconstructions

mPFC lesions were reconstructed onto representative coronal sections from a commercially available atlas of the mouse brain (Paxinos and Franklin, 2008). For each mouse, representative coronal sections from the atlas were aligned with processed images according to structural landmarks local to the area of interest (e.g., the corpus callosum and cell layers for mPFC). For each mouse, regions containing lesioned or damaged tissue were mapped onto the atlas sections using Adobe Illustrator. Lesioned mice that were able to learn were plotted with opacity equal to 33% (given that lesions from three of the mice would overlap at each rostral and caudal target location, such that tissue regions in which all mice showed damage added to an opacity of ∼100%). Lesioned nonlearners were plotted with opacity equal to 20% (given that lesions from 5 mice overlapped at the 2 locations). A similar method was used to reconstruct tdTomato injection sites in the mPFC, cerebellar infusion sites, amygdala infusion sites, and anterogradely labeled terminals in the pons. The locations of retrogradely labeled cells in the pontine nuclei were reconstructed onto representative atlas sections by placing the aligned images over atlas sections and morphing the image to align local structures (such as the pyramidal tract and ventral brain surfaces). Dots were used to mark the locations of labeled soma. In some cases, alignment was imperfect due to differences in the angle of sectioning relative to the atlas. In these cases the relationship between terminal fields and labeled soma were always maintained, despite small inaccuracies in placement relative to local structure.

## RESULTS

### Mice are able to learn and show well timed responses using a trace 50–250 eyeblink conditioning task

To determine whether mice were able to acquire trace eyeblink CRs using a blue light CS while head-restrained on a freely-moving running wheel, we trained mice (*n* = 16) using a trace 50–250 protocol (a 50 ms light CS followed by a 250 ms stimulus-free trace interval, terminating with an air puff US; Fig. 1*B*, top). A 250 ms trace interval was chosen for initial characterization because previous studies indicated that a 250 ms interval was forebrain-dependent in rodents (based on hippocampal lesions; Weiss et al., 1999; Tseng et al., 2004). A relatively short CS was used to keep the total interstimulus interval consistent with that used in previous studies of delay conditioning (200–400 ms; Chettih et al., 2011; Heiney et al., 2014).

#### Acquisition

**Figure 2:**
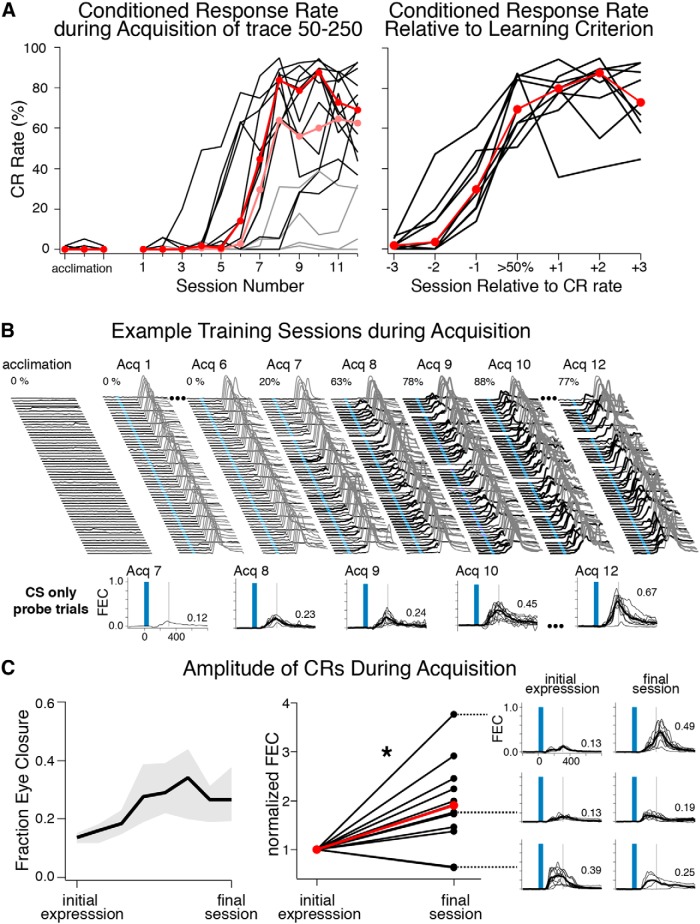
Acquisition rates, example behavior, and CR amplitudes for mice trained using a trace 50–250 protocol. ***A***, CR rates for 16 mice over 12 training sessions. Note that mice showed very low spontaneous blink rates during acclimation sessions, during which no conditioning stimuli were presented (left graph). Most mice (12/16, black lines) learned to a criterion of >50% CRs within 12 sessions (median CR rate each session for learners is shown in red; light gray lines show mice that failed to meet criterion, and light red shows median CR rates for all 16 mice). When aligned to criterion sessions the data show that mice increased expression at similar rates (right graph, red markers denote median CR rates), even though the onset of learning varied across mice. ***B***, Example acclimation and acquisition sessions for one mouse. Like all mice studied here, this mouse showed low spontaneous blink rates during acclimation and initial training sessions (left waterfall plots). Note the expression of CRs reached asymptotic levels by Acq 9 (right waterfall plots), whereas the amplitude of CRs continued to increase over several additional sessions (most clearly observed in overlaid probe trials, bottom graphs). ***C***, Left graph shows the median (black line) and interquartile range (gray shaded area) for the amplitude of CRs measured from probe trials for the 12 learners. Most mice showed significant increases in the amplitude of CRs between initial expression and the last training session (middle graph, paired *t* test, *t* = 2.73, df = 11, *p* = 0.02; examples for 3 mice given in right graphs, including one of the two mice that showed the opposite trend, numbers indicate median amplitude for that session).

Before training began, mice were first given experience on the running wheel (15–20 min for 1–2 d) followed by three baseline acclimation sessions (1 per day) to determine spontaneous blink rates while the mice became familiar with experimental procedures. During this phase, sessions were identical to those used during training except no conditioning stimuli were presented. Mice showed very low spontaneous blink rates during acclimation sessions (0–5%; Fig. 2*A*,*B*, left). With training, 75% of mice learned to express conditioned eyeblink responses (>50% CR rate) within 5–12 sessions, with asymptotic performance rates ranging between 65–95% (Fig. 2*A*, left graph). Conditioned responses were expressed unilaterally (verified in 2 mice, 75% and 85% CRs rates in trained eyes, 0% and 12% CR rates in untrained eyes, data not shown). Critically, startle responses (eyelid responses <50 ms after CS onset) were not observed in any of the mice (Figs. 1*C*, 2*B*). To ensure that the camera sampling rate and analysis was sensitive enough to have detected startle-associated eyelid responses, we presented four mice with the blue light CS (20 trials) followed by trials in which an intentionally startling tone was presented instead (Fig. 1*E*). Startle-associated eyelid responses to the light CS were never observed, even when sampling at 1500 fps, whereas startle responses to the tone were readily observed at 200 fps (Fig. 1*E*). Furthermore, downsampling from 1500 to 187 fps suggested that little if any information was lost at the lower sampling rate. Three of the four mice showed tone-evoked startle that was readily detected by our eyelid analysis at 200 fps (Fig. 1*E*, Mice A–C), whereas the fourth mouse did not show detectable eyelid startle responses even at the higher sampling rate (Mouse D). As a second measure, potential freezing in response to presentation of the light CS was analyzed in six mice in which wheel rotation was measured throughout the first training session. Trials preceded by wheel movement (for the 250 ms prior to CS onset, mean pre-CS speed = 4.4 ± 1.5 cm/s) did not show systematic decreases in speed during the 250 ms following CS onset (mean post-CS speed = 4.3 ± 1.4 cm/s, paired *t* = −1.31, df = 5, *p* = 0.25*^a^*; Fig. 1*D*), indicating that the mice were not freezing to the light CS.

For acquisition of trace 50–250, the number of sessions needed before CRs were observed varied across mice. However, once CRs began to be expressed the rate of learning appeared similar, proceeding gradually over three to five sessions for most mice. To illustrate this, performance across acquisition sessions were aligned to the first sessions in which CR rate exceeded 50%, and shows that from the onset of learning mice increased CR rates by ∼33% per day, and then increased at a rate of ∼9% per day after the 50% CR rate criterion was met and until asymptote was reached (Fig. 2*A*, right; example behavior shown in Fig. 2*B*, top). To demonstrate that the anticipatory eyelid closures reflected associative learning and did not develop in response to repeated light presentations, 8 naïve mice received pseudoconditioning in which the CS and US were never paired (the US alone was presented every fifth trial, and the CS alone was presented on all other trials). Mice failed to develop eyelid responses over 12 sessions (CR rates across all mice and sessions ranged between 0–4.6%, session means across mice ranged between 0–1%; paired *t* test between first and last session, *t* = 0.02, df = 7, *p* = 0.49*^b^*), indicating that the CRs observed in the mice that received paired training reflected associative learning.

#### Response magnitude and timing

Similar to the gradually increasing rate of CRs in the course of learning, the amplitude of CRs (as measured in CS-only probe trials; Fig. 1*C*, bottom; see Materials and Methods) also appeared to increase gradually during acquisition (Fig. 2*B*,*C*, left). Although mice showed asymptotic CR rates within 1–2 sessions after exceeding the 50% learning criterion, CR amplitude increased more gradually over several sessions (Fig. 2*C*, left). Most mice showed significant increases in CR amplitude between initial expression and that observed after 3 or more subsequent training sessions (paired t test, *t* = 2.73, df = 11, *p* = 0.02*^a^*; Fig. 2*C*, right graph; example eyelid responses from 3 mice are also shown for early and postasymptotic sessions). In contrast, the timing of the peak of CRs (taken as the latency to peak amplitude for CS-only probe trials; Fig. 1*C*, bottom) was not different between initial and postasymptotic training sessions (paired t test, *t* = 1.85, df = 11, *p* = 0.09*^b^*), suggesting that the timing of learned responses is fixed early in learning. Furthermore, the latency to the peak amplitude of CRs was well timed to predict the US at the training interval used. At asymptotic performance, the latency to peak of CRs was not different from what would be predicted based on the timing of the US (300 ms after CS onset for trace 50–250: median latency = 297 ms; IQR = 271–329 ms; H_0_: mean = 300 ms, *t* = 0.25, df = 11, *p* = 0.81*^c^*).

**Figure 3:**
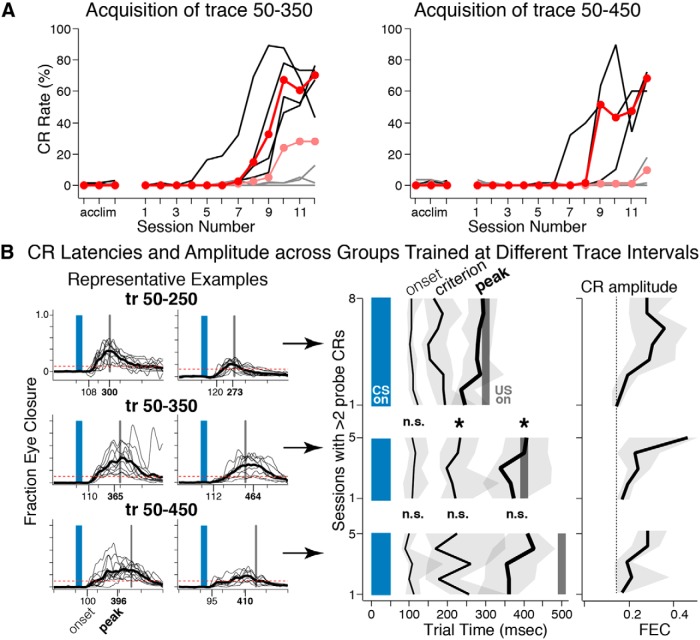
Learning rates and CR latency data for mice trained at trace 50–350 or trace 50–450. ***A***, Only 4 of the 8 mice were able to learn trace 50–350 (left graph), and only 3 of 8 mice were able to learn trace 50–450 (right graph) within 12 training sessions (black lines indicate learners and gray lines indicate nonlearners; red markers show median CR rate for learners and light red markers show the median CR rate for all mice). ***B***, Left graphs show probe trials from two example mice from each of the three groups trained at different trace intervals. Numbers on *x*-axes give the median latency to CR onset and peak for each example mouse. Note that the latencies to onset are similar across these examples, while the latencies to peak shift with the longer training intervals. Right graph shows group data for CR latencies and amplitude for the 3 training groups beginning with initial expression until the last training session (black lines show median latencies, shaded gray region indicates interquartile range). Mice showed CR onsets that did not vary across the different trace intervals on the final training days (trace 50–250 vs 50–350, Wilcoxon rank: *U* = 26, *n* = 4 and 12, *p* = 0.42; trace 50–350 vs 50–450, *U* = 5, *n* = 3 and 4, *p* = 0.69). Latencies to CR criterion and peak were different between trace 50–250 and 50–350 (Wilcoxon rank: *U* = 45, *n* = 4 and 12, *p* < 0.001 and *U* = 44, *p* < 0.001, respectively), with the peaks of CRs appropriately timed for the training intervals used. Mice trained to trace 50–450 showed early latencies relative to the training interval, and were not different from mice trained to 50–350 for any parameter (Wilcoxon rank, Onset: *U* = 5, *n* = 3 and 4, *p* = 0.69; Criterion: *U* = 8, *p* = 0.31; Peak: *U* = 6, *p* = 0.57). CR amplitudes were similar between the three training groups (rightmost graph, black lines show median for each training day, gray shaded area shows interquartile range, dotted line indicates median amplitude of trace 50–250 mice on the first day of expression as reference).

To further investigate the timing of CRs, two sets of naïve mice were trained to different trace intervals (trace 50–350: *n* = 8, and trace 50–450: *n* = 8). Fewer mice were able to learn the longer intervals within 12 training sessions (4/8 mice for trace 50–350 and 3/8 mice for trace 50–450; Fig. 3*A*), suggesting that an interval even 100 ms longer was more difficult for the mice to learn. Of the mice that were able to learn trace 50–350, the latency to the peak amplitude of responses was significantly later than that observed for mice trained with a trace 50–250 protocol (trace 50–350: median = 407 ms; IQR = 357–456 ms; Mann–Whitney–Wilcoxon Rank test, *U* = 44, *n* = 4 and 12, *p* < 0.001*^d^*; Fig. 3*B*, top and middle, “peak”), and was also well timed to the air puff (H_0_: mean = 400 ms, *t* = 0.32, df = 3, *p* = 0.84*^e^*). However, the latency to the onset of CRs (i.e., when the eyelids initially began to close during a given trial; Fig. 1*C*) was not different between mice that learned trace 50–250 versus those that learned trace 50–350 (tr50–250: median = 105 ms, IQR = 99–112 ms; tr50–350: median = 107 ms, IQR = 98–129 ms; *U* = 26, *n* = 4 and 12, *p* = 0.42*^f^*; Fig. 3*B*, top and middle, “onset”). The results are consistent with previous reports of mice trained in delay eyeblink conditioning using different interstimulus intervals, and demonstrate that mice initiated learned responses 100–125 ms after CS onset regardless of the training interval used, whereas timing the peaks of CRs to coincide with the air puff (Chettih et al., 2011; Heiney et al., 2014). Furthermore, a significant shift in the latency at which CR criterion was met (FEC > 0.1; Fig. 1*C*) between trace 50–250 and 50–350 suggests that the difference in the timing of CR peaks was modulated by the initial velocity of behavioral responses (tr50–250: median = 164 ms, IQR = 148–181 ms; tr50–350 median = 234 ms, IQR = 192–266 ms; *U* = 45, *n* = 4 and 12, *p* < 0.001*^g^*; Fig. 3*B*, top and middle, “criterion”). Interestingly, the latencies to onset, criterion and peak amplitude of CRs for mice that learned the trace 50–450 task protocol were not different to those observed for trace 50–350 (tr50–450 Onset: median = 100 ms, IQR = 98–114 ms, *U* = 5, *n* = 3 and 4, *p* = 0.69^h^; Criterion: median = 225 ms, IQR = 223–285 ms, *U* = 8, *p* = 0.31*^i^*; Peak: median = 410, IQR = 403–411 ms, *U* = 6, *p* = 0.57*^j^*; Fig. 3*B*, middle and bottom). The data suggest that the mice able to learn trace 50–450 did so by expressing early CRs that were not well timed to the air puff, and that this interval may be too long for naïve mice to express well timed responses. Similar to trace 50–250, mice trained at longer intervals also initially showed low amplitude CRs at initial expression that gradually increased over several sessions (Fig. 3*B*, right graphs).

### *Mice show long-term retention and flexible learning for trace 50*–*250 eyeblink conditioning*

**Figure 4:**
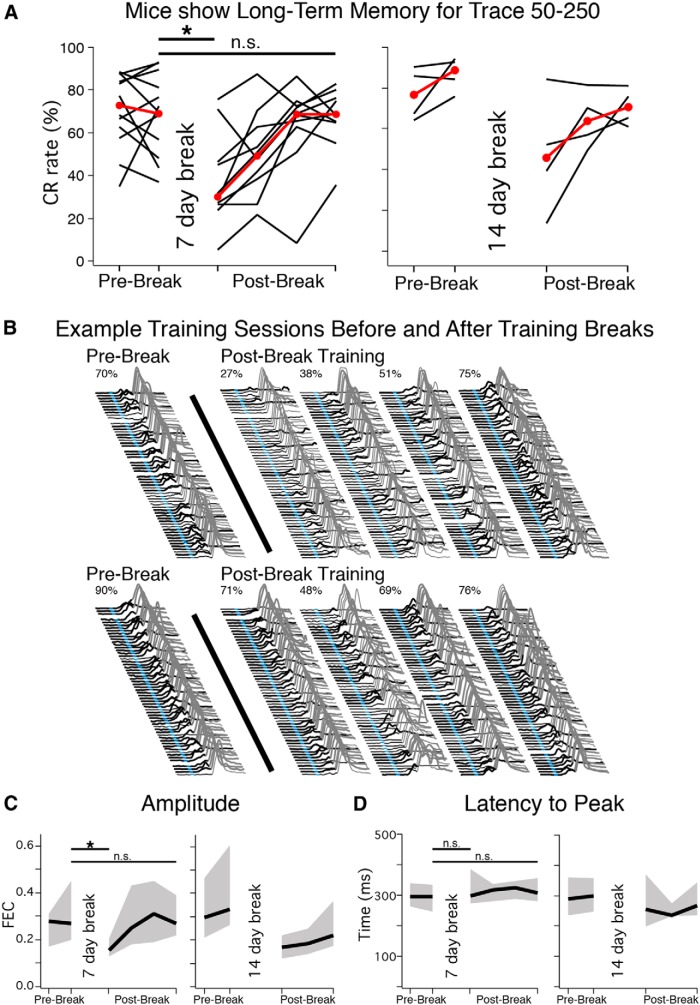
Trained mice receiving a 7 (*n* = 10) or 14 d (*n* = 4) break in training showed long-term memory for the task. ***A***, Asymptotic (Prebreak) CR rates and postbreak performance for mice that received a 7 (left) or 14 d (right) break in training (shown separately but analyzed together). Red markers indicate median observed each day. Mice showed a significant decrease in performance during the first postbreak sessions (paired *t* = 5.69, df = 13, *p* < 0.001), which recovered with additional training sessions (*t* = 1.02, *p* = 0.33). ***B***, Example sessions from two mice before (Prebreak) and after (Postbreak) training breaks. Note that mice showed CRs early in the first postbreak session, even when overall performance was low (top), suggesting that mice remembered rather than quickly relearned the task. ***C***, Similar to CR rate, CR amplitude decreased between prebreak and postbreak sessions (*t* = 3.99, *p* = 0.001), which recovered with additional training (*t* = 0.25, *p* = 0.81). ***D***, In contrast, the appropriate timing for the task (latency to CR peak) was conserved between prebreak and postbreak sessions (*t* = 0.69, *p* = 0.51).

To test the ability of mice to maintain and recall the CS–US relationship, mice trained using the trace 50–250 interval were given a 7 (*n* = 10) or 14 d (*n* = 4) break in training after initial acquisition. The rate of CR expression for the first session after the break was highly variable (7 d median = 30%, IQR = 26–46%; 14 d median = 46%, IQR = 26–68%), but clearly shows that most mice remembered the task (H_0_: mean = 0%, *t* = 6.47, df = 13, *p* < 0.01*^k^*; Fig. 4*A*,*B*; data from the 7 and 14 d training breaks are plotted separately but were not significantly different, Mann–Whitney–Wilcoxon test: *U* = 25, *n* = 4 and 10, *p* = 0.27*^l^*, and so were pooled for testing). In fact, half of the mice showed a CR on the very first training trial, indicating that mice remembered the CS–US relationship rather than rapidly reacquiring the task. Mice with relatively low initial CR rates expressed learned responses intermittently during the entire first postbreak session, rather than gradually increasing the expression of CRs or developing CRs late in the session as would be expected if mice reacquired rather than remembered the task (Fig. 4*B*, top). As noted, however, mice did show a decrease in performance in the first session after the breaks (prebreak: mean = 73±5% CR rate, postbreak: mean = 41±6% CR rate, paired *t* = 5.69, df = 13, *p* < 0.001*^m^*), which recovered after 3–4 d of additional training (+3 sessions: mean = 68±3% CR rate, paired *t* = 1.02, df = 13, *p* = 0.33*^n^*; Fig. 4*A*). The initial decreased performance appeared to be associated with a decrease in CR amplitude (prebreak: mean = 0.36 ± 0.04 FEC, postbreak: mean = 0.20±0.03 FEC; paired *t* = 3.99, df = 13, *p* = 0.001*^p^*), which also recovered with additional training (+3 sessions: mean=0.35±0.05 FEC, paired *t* = 0.25, df = 13, *p* = 0.81*^q^*; Fig. 4*C*, data plotted separately but pooled for testing). In contrast, the timing of CRs (taken as the latency to the peak amplitude of responses during CS-only probe trials) was not affected by the break in training (prebreak: mean=294 ± 13 ms, postbreak: mean = 324 ± 38 ms; paired *t* = 0.69, df = 13, *p* = 0.51*^r^*; Fig. 4*D*). The data suggest that mice can show long-term retention of the CS–US relationship and the timing of CRs.

**Figure 5:**
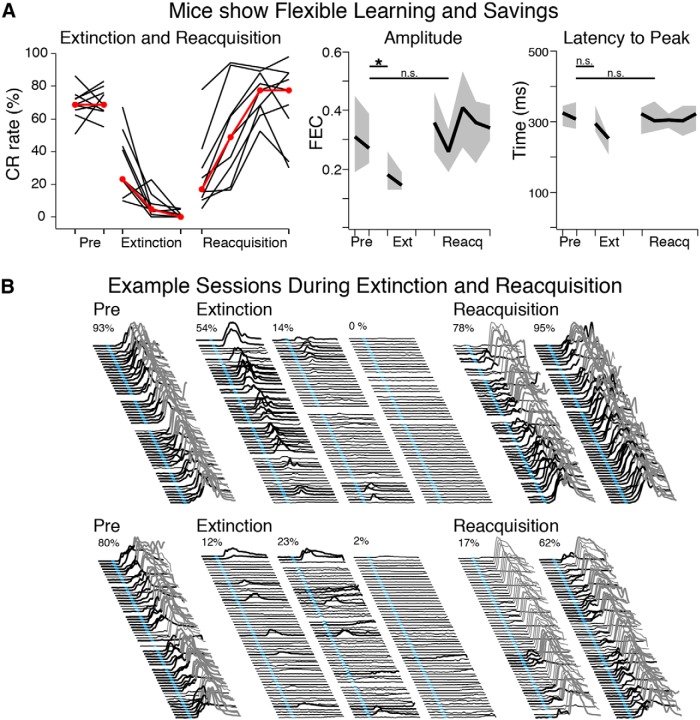
Mice efficiently extinguished learned responses, and showed savings during reacquisition. ***A***, CR rates for mice during asymptotic performance (Pre), and for extinction and reacquisition sessions. Mice (*n* = 9) decreased CR rates to <5% within three extinction sessions, and reacquired CRs faster than during initial acquisition (“savings”; left graph, red markers show median CR rate for each session). Decreases in CR rate were accompanied by decreases in amplitude (middle graph, median ± IQR, *t* = 3.21, df = 8, *p* = 0.01), but not by differences in CR peak latencies (right graph, median ± IQR, *t* = 0.10, *p* = 0.92). ***B***, Example extinction and reacquisition sessions from two mice. For extinction, some mice decreased CRs gradually (top) while others showed more abrupt changes in behavior (middle). Note the decrease in CR amplitude during extinction sessions, whereas the timing of CRs was maintained. All mice met learning criterion (>50% CRs) within one to three reacquisition sessions. Some mice showed gradual learning during a session (top), whereas others increased expression between sessions (bottom).

Mice also displayed flexible learning. Mice learned not to respond during sessions in which the light was no longer paired with the air puff (i.e., extinction training), with CR expression fully extinguished in all mice by the third day (0–5% CRs, *n* = 9, Fig. 5*A*, left). The CRs expressed by some mice extinguished slowly, with gradually decreasing CR amplitudes (Fig. 5*B*, top), whereas the CRs of other mice extinguished quickly, after only a few CS-only trials (Fig. 5*B*, bottom). In general, extinction appeared to be associated with a decrease in CR amplitude (pre-extinction mean = 0.36 ± 0.06 FEC, extinction day 1 mean = 0.21 ± 0.02 FEC; paired *t* = 3.21, df = 8, *p* = 0.01*^s^*; Fig. 5*A*, middle graph), whereas the timing of CRs was not different than before extinction training (pre-extinction mean = 307 ± 6 ms, extinction day 1 mean = 308 ± 11 ms; paired *t* = 0.10, df = 8, *p* = 0.92*^t^*; Fig. 5*A*, right graph). When paired trials were reinstated (reacquisition training), all mice showed behavioral savings and reacquired CRs much faster than original acquisition (within 1–3 sessions; Fig. 5*A*, left, B). By the second reacquisition session mice showed similar CR amplitudes and timing relative to pre-extinction training (Amplitude: reacquisition mean = 0.37 ± 0.09 ms, paired *t* = 0.17, df = 7, *p* = 0.87*^u^*; Latency to CR Peak: reacquisition mean = 303 ± 16 ms, paired *t* = 0.18, df = 7, *p* = 0.86*^v^*; one mouse did not show sufficient CRs during probe trials to calculate median measures and was not included in the latter comparisons; Fig. 5*A*, middle and right graphs). The data show that in addition to learning a trace 50–250 eyeblink conditioning task, mice can also learn not to respond and that the learned behavior can be efficiently reinstated through experience.

### *Trace 50*–*250 is dependent on the mPFC in mice*

**Figure 6:**
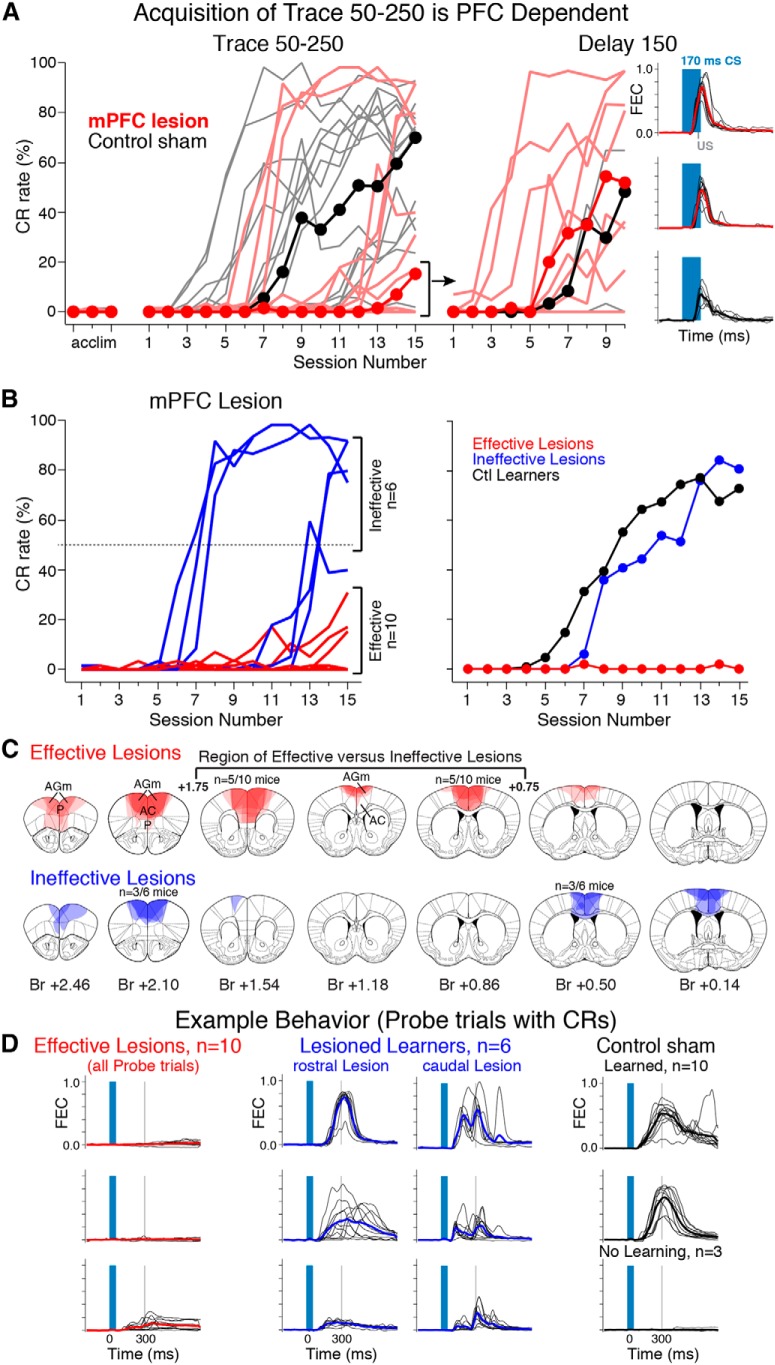
Acquisition of trace 50–250 is dependent on a subregion of mPFC in mice. ***A***, CR rates during acclimation and training sessions (left graph) in mPFC-lesioned (light red lines) and sham-operated control mice (light black lines). Black and red markers show median CR rates observed each session for control and lesioned mice, respectively. Note that although a subset of lesioned mice learned, the median value indicates that most lesioned mice showed few if any CRs after 15 training days. Most lesioned nonlearners were able to acquire normal CRs for a non-mPFC-dependent delay eyeblink-conditioning task, showing that the deficit was specific to trace conditioning and did not reflect a global learning impairment in these mice (right graph, example behavior from 2 lesioned and 1 control mice are shown, denoted by red and black lines, respectively). ***B***, CR rates for all lesioned mice, color-coded as lesioned nonlearners (red lines; Effective) and lesioned learners (blue lines; Ineffective). Median CR rates between control mice (right graph, black markers) and mice with ineffective mPFC lesions (blue markers) were similar, in contrast to mice that did not learn the task (red markers). ***C***, Lesion reconstructions for all mPFC lesioned mice, separated into Effective (red, top row; *n* = 10) and Ineffective (blue, bottom row; *n* = 6) mice (opacity was adjusted for each mouse such that the total number of mice = 100%). Note that mice with damage to at least 500 µm of the medial agranular (AGm) and anterior cingulate (AC) between bregma +0.75–1.75 were not able to acquire the task (top, red shaded regions within bracketed sections), whereas mice with lesions that spared this area were able to learn (bottom). ***D***, Example behavior from probe trials from effective lesions (red), ineffective lesioned learners (blue), and control mice (black). Most lesioned mice showed no learning (red, top 2 graphs). One lesioned mouse that showed some learning but did not meet criterion is also shown (red, bottom graph). Mice with lesions restricted to the most rostral mPFC (anterior to bregma +1.75) showed normal learning (blue, middle left graphs), whereas lesions restricted to the most caudal mPFC (posterior to bregma +0.75) showed abnormal CRs with uncharacteristic double-peaked responses (blue, middle right graphs) relative to control mice (black, right graphs). One example from a control mouse that did not learn is also shown (bottom right graph). Br, Bregma; AGm, medial agranular cortex; P, prelimbic cortex; AC, anterior cingulate cortex.

We next tested whether the 250 ms trace interval was indeed forebrain-dependent under these training conditions. Sixteen naïve mice received mPFC lesions before training. Half received lesions targeted rostral to the genu of the corpus callosum (bregma +1.5–2.5) and half received lesions targeted caudal to the genu (bregma +0.5–1.5). Sixteen corresponding control mice for the two groups received craniotomies but no tissue was aspirated. For sham control mice, 10/16 mice learned to a criterion of >50% CR rate within 15 sessions. Three mice that failed to meet this criterion showed some learning (20–45% CR rate), and the remaining three showed few or no CRs (<5%). In contrast, only 6/16 lesioned mice were able to learn the task. One lesioned mouse that did not meet the 50% criterion showed some learning (31% CR rate), however the majority of lesioned mice showed little or no learning (9/16 mice, 0–15% CR rate; Fig. 6*A*, Trace 50–250, left graph; median CR rate is shown for each training day between lesioned and sham controls to highlight that most lesioned mice overlap at 0% CR rates). The data for mPFC lesioned mice are shown separately in Figure 6*B* (left graph) to highlight the difference in learning curves between lesioned nonlearners (“Effective”, red lines) versus lesioned learners (“Ineffective:, blue lines). The acquisition rates of mice with ineffective lesions appeared similar to that observed for control learners across training days (Fig. 6*B*, right graph, compare blue to black symbols; effective lesion data are also shown for comparison).

Of considerable interest was the anatomical distribution of effective and ineffective lesions. Lesion reconstructions revealed that the locations of mPFC damage were clearly different between lesioned learners and nonlearners, allowing for the critical region of the mPFC for trace conditioning to be demarcated. All mice with substantial lesions between bregma +1.75 and +0.75 were unable to learn the task (Fig. 6*C*, top middle, D, left). Seven of the 10 mice with these more central lesions had a CR rate of 0% on their 15^th^ training session. In contrast, mice with lesions that were restricted to the more rostral mPFC (between bregma +1.75 and +2.75) were able to learn normally (Fig. 6*C*, bottom left, D, compare middle left examples to controls). Likewise, mice with lesions restricted to the more caudal mPFC (between bregma 0.0 and +0.75) were also able to learn (Fig. 6*C*, bottom right), although the CRs of these mice had an abnormal topography with double peaked responses that were never observed in other mice (Fig. 6*D*, compare middle right examples to controls). Importantly, 8/9 of the mice with the more mid-range mPFC lesions that prevented the acquisition of trace conditioning proceeded to show normal learning in the forebrain-independent delay version of the task (delay 150, CR rates: 5/9: >50%, 3/9: 17–39%; Fig. 6*A*, right; note that 1 sham control mouse also did not learn the delay task). These data suggest the learning deficit observed for Trace 50–250 was specific to the forebrain-dependent version of the task, and did not represent a generalized learning deficit. Furthermore, the data show that disruption of as little as ∼500 μm of the AGm and ACc regions of the mPFC between bregma +0.75 and +1.75 (i.e., a 1 mm range centered around the genu) prevents the acquisition of trace eyeblink conditioning in mice.

**Figure 7:**
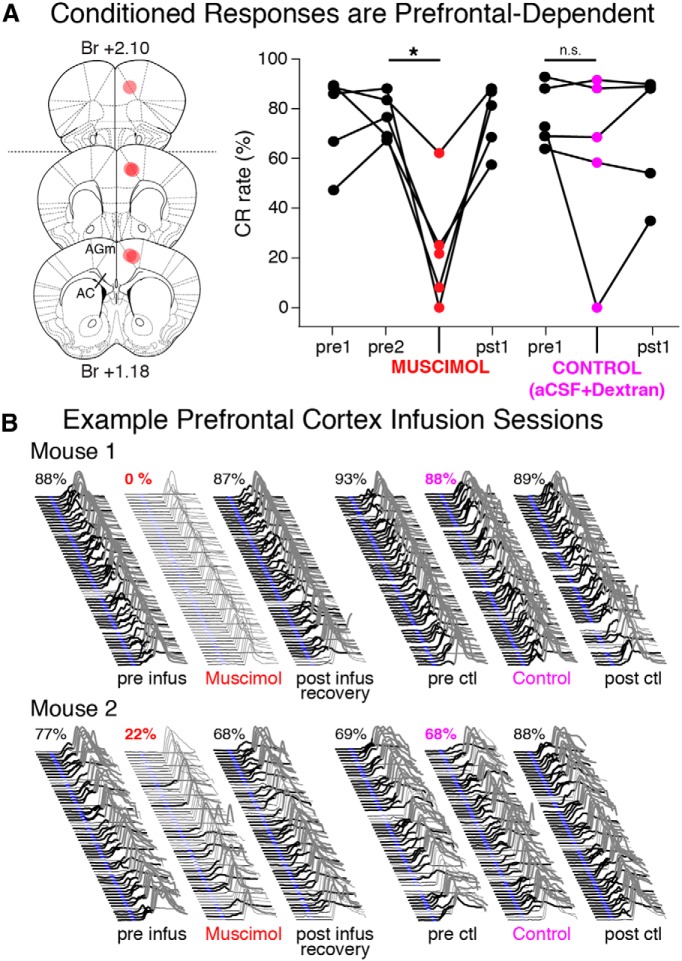
Trace 50–250 CRs were prefrontal-dependent in mice trained to asymptotic performance. ***A***, Unilateral infusions of muscimol (1mm, 100–125 nl) were used to inactivate the mPFC of 5 trained mice (left). Inactivation of the mPFC resulted in reliable decreases in the expression of CRs in trained animals (right graph, red markers; paired *t* = 4.86, df = 5, *p* = 0.008), whereas the CR rates observed during control infusions were not different than preinfusion sessions (magenta markers; *t* = 1.13, *p* = 0.32). ***B***, Example behavior from muscimol (denoted by red text) and control sessions (magenta text) for two mice. Note the significant decrease in the expression of CRs during muscimol infusion sessions, while behavior during control sessions was less affected by the infusion procedure. Br, Bregma; AGm, medial agranular cortex; AC, anterior cingulate cortex.

The lesion experiment indicated that a restricted region of caudal mPFC supports the acquisition of trace eyeblink conditioning in mice. To test whether the same region of mPFC also supports postacquisition expression of trace CRs, we used unilateral infusions of muscimol (100–125 nl) to temporarily inactivate this region of mPFC after training to asymptotic performance (5/8 mice learned, preinfusion mean = 77 ± 4% CR rate). Inactivation resulted in significant decreases in the expression of CRs in 4/5 mice (infusion session mean = 23 ± 11% CR rate, paired *t* = 4.86, df = 4, *p* = 0.008*^w^*; Fig. *A*, right; example behavioral sessions given in B, left). As a control to ensure that the infusion procedures alone did not affect the expression of CRs, mice also received infusions of Alexa-conjugated dextran amines dissolved in aCSF (100–125 nl; used to better visualize infusion sites) using the same procedures. Control infusions did not reliably affect the expression of CRs (precontrol infusion mean = 77 ± 6% CR rate, control infusion mean = 61 ± 17% CR rate; paired *t* = 1.13, df = 4, *p* = 0.32*^x^*; Fig. 7*A*, right; examples in B, right). Histology revealed that the infusion site of one mouse that showed only a modest decrease in the expression of trace CRs during inactivation was located anterior to the critical region identified in the lesion study (Fig. 7*A*, left, dashed line indicates anterior border of critical region). The control infusion of a second mouse was unusual in that it resulted in an abolishment of CRs. The histology from this mouse revealed substantial damage at the infusion site, apparently due to a blocked cannula that released a bolus of dextran when positioned. The remaining histology confirmed infusion sites within the critical region of mPFC with minimal damage. The data show that the critical region of mPFC necessary for the acquisition of trace CRs also supports postacquisition expression.

### Trace 50–250 learned responses are completely abolished during cerebellar inactivation

**Figure 8:**
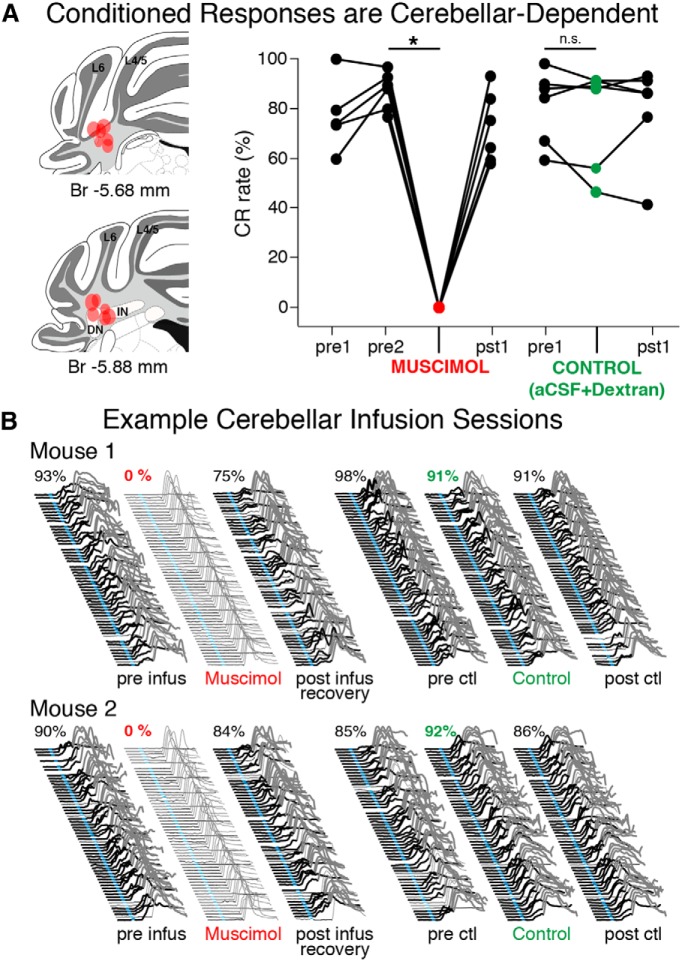
Trace 50–250 CRs were cerebellar-dependent in mice, with no residual noncerebellar behavioral components. ***A***, Infusions of muscimol (1mm, 100–150 nl) were used to inactivate anterior cerebellar regions in 6 trained mice (left). The expression of CRs were completely abolished during infusion sessions in all 6 mice (right graph, red markers; paired *t* = 28.11, df = 5, *p* < 0.001), whereas the CR rates observed during control infusions were not different than preinfusion sessions (green markers; *t* = 1.07, *p* = 0.34). ***B***, Example behavior from muscimol (denoted by red text) and control sessions (green text) for two mice. Note the complete absence of CRs during muscimol infusion sessions, whereas behavior during control sessions was largely unaffected by the infusion procedure. Br, Bregma; L6, cerebellar cortex lobule 6; L4/5, cerebellar cortex lobule 4/5; DN, dentate nucleus; IN, interpositus nucleus.

We tested the cerebellar-dependence of learned responses for trace 50–250 by infusing muscimol into anterior cerebellar regions previously shown to support eyeblink conditioning (at the border of cerebellar lobules 4/5 and 6, and/or the anterior interpositus/dentate nuclei; Fig. 8*A*, left; McCormick et al., 1982; Yeo et al., 1985a,b; Plakke et al., 2007; Kalmbach et al., 2010a; Gonzalez-Joekes and Schreurs, 2012; Heiney et al., 2014). After mice were trained to asymptotic performance (6/8 implanted mice learned, preinfusion mean = 87 ± 3% CR rate), eyeblink-associated cerebellar regions were inactivated by infusing muscimol (100–150 nl) into the cerebellum 15 min before a training session began. Inactivation resulted in the complete abolishment of CRs in all six cases (infusion session mean = 0 ± 0% CR rate, paired *t* = 28.11, df = 5, *p* < 0.001*^y^*; Fig. 8*A*, right, example behavioral sessions given in B, left). As a control to ensure that the infusion procedures alone did not affect the expression of CRs, mice also received control infusions of Alexa-conjugated dextran amines dissolved in aCSF (100–150 nl; used to better visualize infusion sites and for anatomical tracing, see below) using the same procedures. The expression of CRs following control infusions was not different from that observed during a typical training session (precontrol infusion mean = 81 ± 6% CR rate, control infusion mean = 77 ± 8% CR rate; paired *t* = 1.07, df = 5, *p* = 0.34*^z^*; Fig. 8*A*, right, examples given in B, right). The data show that the entire learned motor response was cerebellar-dependent, and did not include a noncerebellar component as previously observed in mice (Koekkoek et al., 2005; Sakamoto and Endo, 2010).

### *Trace 50*–*250 learned response rates reliably decrease during amygdala inactivation*

**Figure 9:**
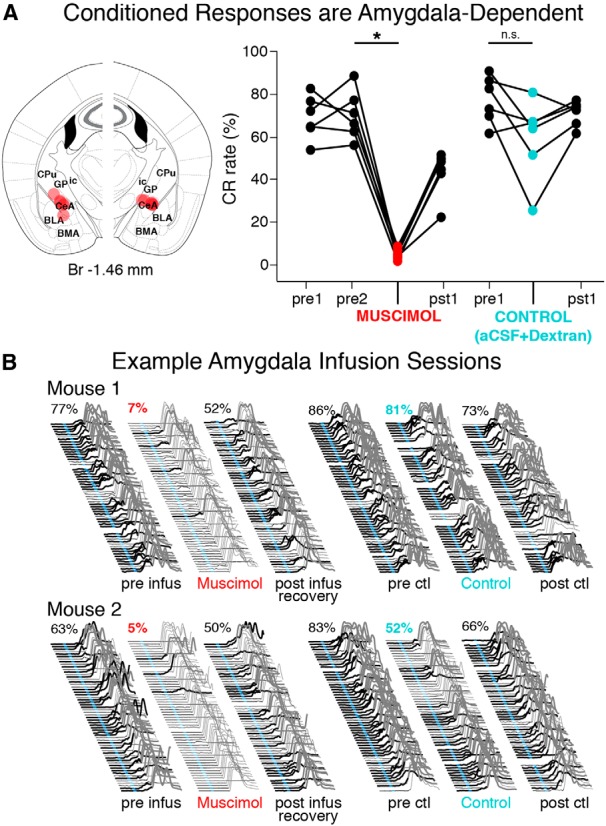
Trace 50-250 CRs were amygdala-dependent in mice, with no residual CR components. ***A***, Bilateral infusions of muscimol (1mm, 100–150 nl) were used to inactivate the anterior central nuclei of the amygdala in 6 trained mice (left). The expression of CRs were nearly abolished (2–9% CR rates) during infusion sessions in all 6 mice (right graph, red markers; paired *t* = 14.27, df = 5, *p* < 0.001), whereas the CR rates observed during control infusions were not different than preinfusion sessions (cyan markers; *t* = 2.34, *p* = 0.07). ***B***, Example behavior from muscimol (denoted by red text) and control sessions (cyan text) for two mice. Note the significant decrease in the expression of CRs during muscimol infusion sessions, while behavior during control sessions was less globally affected by the infusion procedure. Br, Bregma; CeA, central nucleus of the amygdala; BLA and BMA, basolateral and basomedial nuclei of the amygdala, respectively; CPu, caudate putamen; GP, globus pallidus; ic, internal capsule.

In addition to sometimes driving a noncerebellar learned eyelid response (see Introduction), it has also been suggested that the amygdala may facilitate other inputs to the pons and enhance the neural representation of the CS before being projected to the cerebellum. As a follow-up to the cerebellar inactivation experiment, we tested the amygdala-dependence of learned responses for trace 50–250 by infusing muscimol into the central nucleus after training to asymptotic performance (6/7 implanted mice learned, preinfusion mean = 70 ± 5% CR rate). Inactivation resulted in significant decreases in the expression of CRs in all six mice (infusion session mean = 5 ± 1% CR rate, paired *t* = 14.27, df = 5, *p* < 0.001*^y^*; Fig. 9*A*, right, example behavioral sessions given in B, left). Control infusions did not reliably affect the expression of CRs using the same procedures (precontrol infusion mean = 77 ± 5% CR rate, control infusion mean = 59 ± 8% CR rate; paired *t* = 2.34, df = 5, *p* = 0.07*^z^*; Fig. 9*A*, right; examples given in B, right). Because the CRs in our trained mice are dominated by a well timed response that is reminiscent of the previously reported cerebellar component (Koekkoek et al., 2005; Sakamoto and Endo, 2010), and given the known hodology, we interpret these results as suggesting that the amygdala, like the mPFC and primary sensory regions, may provide a CS-associated input to the cerebellum that supports the learning and ongoing cerebellar expression of CRs.

### Pontine cells retrogradely labeled from cerebellar regions of functional inactivation overlap with mPFC and amygdala terminals in the basilar pons

**Figure 10:**
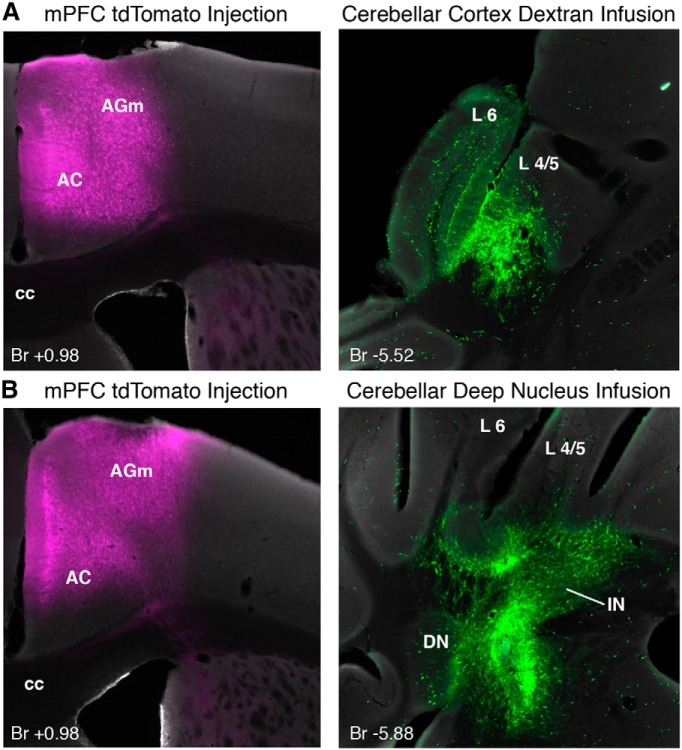
Injections of AAV to express td Tomato in the mPFC and infusions of AlexaFluor-conjugated dextran in the cerebellum (3000 MW) were used to investigate regions of overlap between mPFC axon terminals and retrogradely labeled cerebellar-projecting neurons. ***A***, Example tdTomato expression in the mPFC (left) of a mouse that received a dextran infusion that overlapped with the anterior cerebellar cortex (right). ***B***, Example tdTomato expression in the mPFC (left) of a mouse that received a dextran infusion that overlapped with the cerebellar deep nuclei (right). Br, Bregma; AGm, medial agranular cortex; AC, anterior cingulate cortex; cc, corpus callosum; L6, cerebellar cortex lobule 6; L4/5, cerebellar cortex lobule 4/5; DN, dentate nucleus; IN, interpositus nucleus.

To further define the putative functional pathway between the mPFC and amygdala with cerebellar eyeblink regions in mice, we examined pontine regions for overlap between mPFC and amygdala terminal fields with cells that were retrogradely labeled during cerebellar control infusions (*n* = 6 mice in which muscimol infusions abolished CRs; Fig. 8*A*). To this end, mice receiving the chronically implanted cerebellar cannula were also injected in the mPFC with a rAAV to express the fluorescent protein tdTomato in prefrontal neurons and their axon terminals. The mPFC injections were targeted to the identical regions deemed necessary for acquisition of trace 50–250 from our lesion experiment (between bregma +1.75 and +0.75; Fig. 10*A*,*B*, left). Amygdala central nucleus terminal fields were anterogradely labeled with AlexaFluor-conjugated dextran during control infusions (*n* = 6 mice in which muscimol infusions disrupted CR expression; see Fig. 12*A*).

#### Medial PFC projection patterns in pontine regions

**Figure 11:**
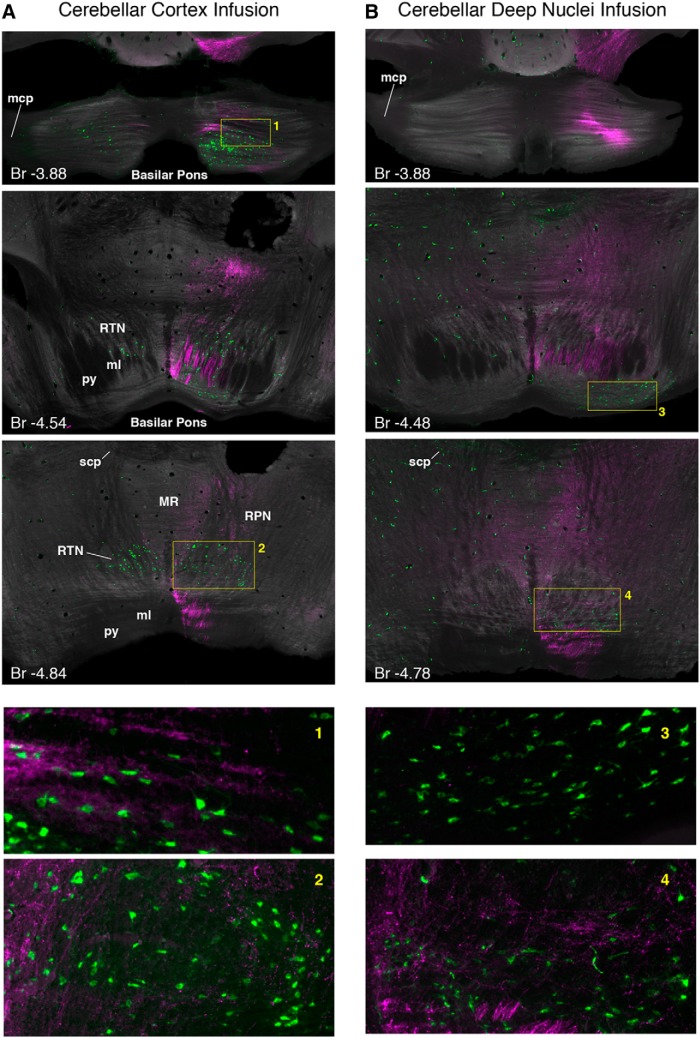
Example histology showing overlap between mPFC terminals anterogradely labeled with td Tomato and retrogradely labeled eyeblink-associated cerebellar neurons. ***A***, Mice that received cerebellar cortex infusions showed retrogradely labeled neurons that extended throughout the anterior–posterior basilar pons (green cells, top 2 images) and into and throughout the RTN (2^nd^ and 3^rd^ images). Substantial overlap between mPFC terminals (magenta) and putative eyeblink-associated cells (green) was observed in the anterior basilar pons (top image, inset 1) and extensively in the RTN (2^nd^ and 3^rd^ images, inset 2). ***B***, Mice that received deep nuclei infusions showed retrogradely labeled neurons (green cells) restricted to the caudal basilar pons (2^nd^ image) and less dense labeling in the more anterior RTN (3^rd^ image). Few if any mPFC terminals were observed in the caudal basilar pons and did not appear to overlap substantially with the labeled pontocerebellar neurons observed there (2^nd^ image, notice the general absence of magenta terminal labeling in inset 3). Overlap was observed the RTN (3^rd^ image, inset 4). Br, Bregma; mcp, middle cerebellar peduncle; ml, medial lemniscus; py, pyramidal tract; scp, superior cerebellar peduncle; MR, median raphe nucleus; RPN, reticular nucleus of the pons.

Anterogradely labeled tdTomato-expressing mPFC terminal fields were observed in the basilar pons and reticulotegmental nucleus (RTN) in all six injected mice, and showed similar projection patterns (e.g., injection sites: Figs. 10*A*,*B*; terminal label: 11*A*,*B*; group summary: 13*A*,*B*). Densely labeled axons were observed in the medial lemniscus ipsilateral to the mPFC injection site, exiting the white matter bundle and forming a dorsal ring of terminal fields around the medial region of the rostral basilar pons and extending into lateral regions (Figs. 11*A*, top; summary 13*B*, left). A small proportion of axons crossed the midline and formed less dense terminal fields in the pons contralateral to the mPFC injection site (Figs. 11*A*, top; summary 13*B*, left). Prefrontopontine terminal fields were most dense and stereotyped across the six mice in the anterior third of the basilar pons, with different mice showing unique restricted and less dense terminal fields in the caudal half of the pons (Fig. 13*B*). However, labeled axons were observed throughout the anterior–posterior extent of the medial lemniscus (Fig. 11*A*,*B*). Extensive mPFC terminal labeling was also observed in the RTN, and was most dense near the midline and in the pericentral region, although labeled terminals were observed throughout most of the structure and surrounding regions (Figs. 11*A*,*B*; summary 13*B*). It was unclear, however, whether the source of terminal fields in the RTN included the medial lemniscus, or whether some or all axonal projections may have originated from the adjacent reticular pontine nucleus or from the median raphe, both of which showed extensive mPFC terminal labeling (Fig. 13*A*,*B*). The data show that the mPFC of mice projects to the anterior basilar pons and throughout the RTN.

#### Amygdala central nucleus projection patterns in pontine regions

**Figure 12:**
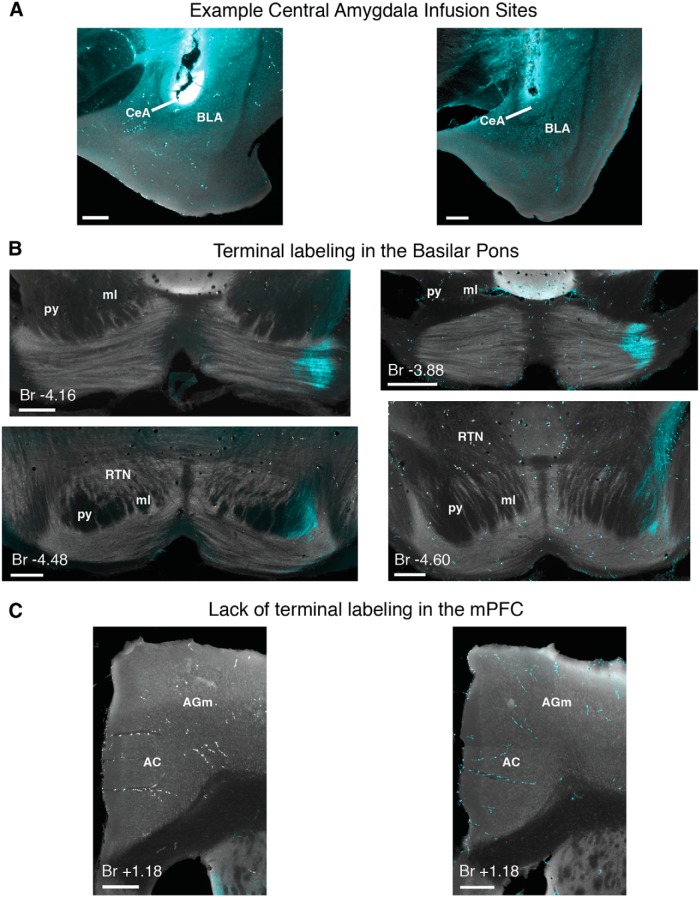
Example histology from two mice showing infusion sites targeting the central nucleus of the amygdala, the distribution of amygdala terminals in the basilar pons, and a lack of substantial label in the RTN and mPFC. ***A***, Infusions of Alexa-conjugated dextran included the central nucleus in both cases. ***B***, Amygdala terminal labeling was observed primarily in the anterior basilar pons, with sparse terminals observed in the caudal basilar pons and RTN. ***C***, Terminal labeling as a result of amygdala central nucleus infusions was not observed in the region of mPFC critical for trace eyeblink conditioning. Br, Bregma; CeA, central nucleus of the amygdala; BLA, basolateral nuclues of the amygdala; ml, medial lemniscus; py, pyramidal tract; AGm, medial agranular cortex; AC, anterior cingulate cortex. Scale bar, 250 µm.

**Figure 13:**
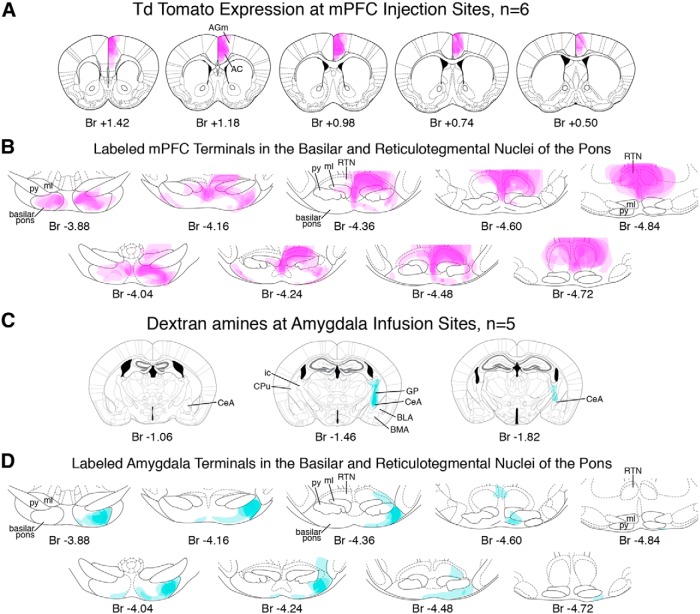
Summaries of mPFC (magenta) and amygdala (cyan) terminal distribution in the basilar pons and RTN. **A.** Reconstructions of td Tomato expressing mPFC cells (*n* = 6 mice, opacity for each mouse adjusted such that overlap of all mice = 100%). Note that the greatest overlap in expression was largely restricted to mPFC regions that are necessary for acquisition of trace eyeblink conditioning (compare with Fig. 6*C*). ***B***, Reconstructions of mPFC terminal labeling in the basilar pons and RTN (opacity for each mouse adjusted such that overlap of all mice = 100%). The greatest overlap was observed in the anterior basilar pons (left sections), and throughout the RTN and surrounding brain regions (right sections). ***C***, Reconstructions of AlexaFluor-conjugated dextran infusions targeting the amygdala central nucleus (*n* = 5 mice, opacity adjusted such that all mice = 100%). The highest concentrations of dextran deposit overlapped with the central nucleus in all cases. ***D***, Reconstructions of central amygdala terminal labeling in the basilar pons (opacity adjusted such that all mice = 100%). The greatest overlap across animals was observed in the anterior basilar pons (left sections), whereas few terminals were observed in the RTN (right sections).

Anterogradely labeled terminal fields were observed in the basilar pons in 5/6 mice (one mouse showed no labeled axons distributed around the infusion site, suggesting a lack of dextran uptake in that mouse). The five mice showed similar projection patterns in the basilar pons (e.g., injection sites: Figs. 12*A*; terminal label: 12*B*, group summary: 13*C*,*D*). Densely labeled axons were observed in the most lateral region of the lateral lemniscus ipsilateral to the amygdala infusion site (Fig. 12*B*). Similar to the pattern of terminal label observed for the mPFC, terminals were most dense at the rostral-most extent of the lateral basilar pons, exiting the white matter bundle and extending to a ventral ring of terminal fields in the around the medial region of the rostral basilar pons (Figs. 12*B*, top; summary 13*D*, left). Few if any axons were observed to cross the midline, with terminal fields being more strictly unilateral than observed for mPFC (Figs. 12*B*, top; summary D, left). Amygdala terminal fields were most dense and stereotyped across mice in the anterior third of the basilar pons (Fig. 13*D*), even though labeled axons were observed throughout the anterior–posterior extent of the lateral lemniscus (Fig. 12*B*). In contrast to mPFC terminal labeling, very few terminal fields were observed in the RTN (summary Fig. 13*D*). The data show that the central amygdala of mice projects most densely to the rostral basilar pons, to regions that overlap with mPFC terminal fields in the most anterior pons region. It should be noted that our amygdala infusions resulted in very few (if any) labeled terminals in the dorsal-caudal mPFC in any of the mice (Fig. 12*C*), suggesting that direct interactions between the central nucleus and the mPFC region critical for trace eyeblink conditioning is unlikely.

#### Identification of pontine cells projecting to eyeblink-associated regions in the cerebellum

In 3 of 6 mice, cerebellar infusions were primarily located rostral to the deep cerebellar nuclei and clearly overlapped with the ventral bank surrounding the border of cerebellar cortex lobules 4/5 and 6 (Figs. 11*A*, right; summary 14*A*). The spread of the retrograde tracer did not appear to extend caudally into the deep nuclei, and was contained within the large bundle of white matter that separates the two regions. Labeled axons were observed in the white matter around the infusion site (Fig. 10*A*, right), but few notable terminals or soma were observed in the deep nuclei in these mice, suggesting that fibers of passage did not take up the tracer. For the remaining three mice the infusion sites were more caudal and within the vicinity of the anterior deep nuclei, in the interpositus and/or dentate regions (Figs. 10*B*, right; summary14*C*). The apparent spread of the dextran in those animals did not appear to reach the overlying and/or more rostrally located cerebellar cortex, although axonal label was clearly seen in those regions suggesting that the axon terminals of Purkinje cells in the deep nuclei took up the tracer and were retrogradely labeled (Fig. 10*B*, right). For both cerebellar cortex and deep nuclei infusions, the substantial number of passing fibers in the intervening white matter did not appear to take up the retrograde tracer, given that significant labeling was not observed over the vast extent of the cerebellar cortex as would be expected (Fig. 10*A*,*B*, right).

**Figure 14:**
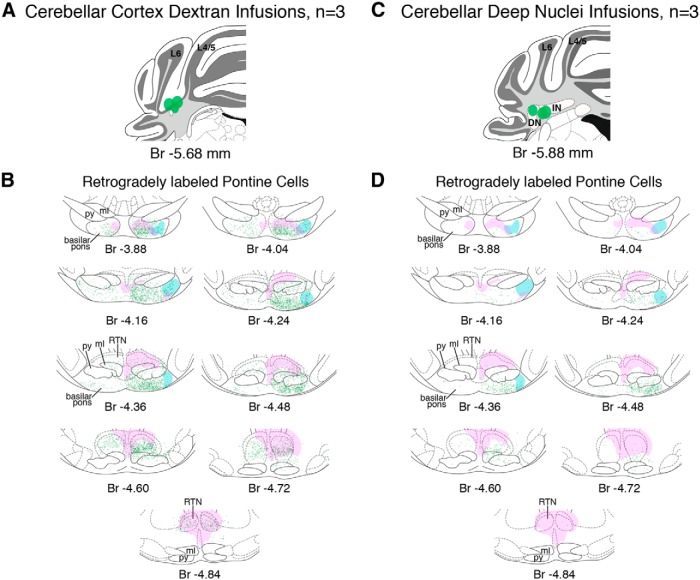
Distribution of cerebellar cortex (*n* = 3) or deep nuclei projecting cells (*n* = 3) and regions of potential interaction between mPFC, amygdala inputs and eyeblink-associated pontocerebellar cells. ***A***, Dextran infusion sites from three mice overlapped with the anterior cerebellar cortex, at the border of lobules 4/5 and 6 around the ventral bank. ***B***, Retrogradely labeled neurons resulting from cerebellar cortex infusions were observed throughout the anterior–posterior basilar pons (top to middle sections; green markers represent each putatively labeled soma) and into and throughout the RTN (middle to bottom). Magenta and cyan shaded regions represent mPFC and amygdala terminal labeling observed in more than half of the mice (4/6 and 3/5, respectively). Substantial overlap between mPFC and amygdala terminals was observed in the anterior basilar pons, both of which overlapped with labeled eyeblink-associated pontocerebellar neurons. ***C***, Dextran infusion sites from three mice overlapped with the anterior dentate and/or anterior interpositus deep cerebellar nuclei. ***D***, Retrogradely labeled neurons resulting from cerebellar deep nuclei infusions were restricted to the caudal basilar pons (middle sections) and in the anterior RTN (middle to bottom). Substantial overlap of these labeled pontocerebellar neurons with mPFC terminals was limited to the RTN of deep nuclei infused mice, in which amygdala terminals were not observed. Br, Bregma; py, pyramidal tract; ml, medial lemniscus; L6, cerebellar cortex lobule 6; L4/5, cerebellar cortex lobule 4/5; DN, dentate nucleus; IN, interpositus nucleus.

The most extensive retrograde labeling in pontine regions was observed in mice as a result of cerebellar cortex infusions. A relatively dense population of labeled somata was observed bilaterally in the RTN, located dorsal to the caudal basilar pons and pyramidal tract and extending >300 μm posterior throughout the extent of that structure (bregma −4.60 to −4.84; Figs. 14*B*, 11*A*). Labeled somata were also observed in the caudal basilar pons (reported to receive primarily sensory inputs in rodents; Wiesendanger and Wiesendanger, 1982; Leergaard and Bjaalie, 2007), which extended along the entire anterior–posterior axis into the rostral basilar pons (bregma −3.88 to −4.48; Figs. 14*B*, 11*A*). Labeling in the basilar pons was also bilateral but much denser in the pons contralateral to the cerebellar infusion site. The axons of labeled pontocerebellar cells were observed crossing the midline of the pons and entering the middle cerebellar peduncle ipsilateral to the infusion site (Fig. 11*A*, top). At the level of the rostral pons, most labeled somata were concentrated in the medial region (a putative region of cerebral motor cortex inputs in rodents; Wiesendanger and Wiesendanger, 1982; Leergaard and Bjaalie, 2007), with more sparsely labeled somata observed in the dorsal and lateral regions of the rostral pons (bregma −3.88 to −4.16; Figs. 14*B*, 11*A*).

The three mice that received infusions of retrograde tracer into the anterior deep nuclei showed similar patterns of somata labeling in the RTN, but appeared less dense than observed for cerebellar cortex infusions and did not extend as far posterior in the structure (∼150 μm; bregma −4.60 to 4.84; Figs. 14*D*, 11*B*). Labeled cells were also observed in the caudal basilar pons, almost exclusively on the side contralateral to the infusion site (bregma −4.36 to −4.48; Fig. 14*D*, 11*B*). However, the density of labeled somata decreased towards the anterior extent, such that few labeled cells were observed in the middle to rostral basilar pons (bregma −3.88 to −4.16; Figs. 14*B*, 11*B*). The source of axons from the labeled cells in the RTN and basilar pons was difficult to identify. In contrast to cerebellar cortex injections, no axon labeling was observed in the middle cerebellar peduncle at this anterior–posterior level or crossing the midline of the pons at any point along the anterior–posterior axis (Fig. 11*B*, top). It should be noted that labeled axons were observed in the superior cerebellar peduncle (which is comprised of deep nuclei efferents) in 2 of the 3 mice, which was not observed in the mice receiving putative cerebellar cortex infusions ( Figs. 11*A*, Br −4.84 compare with 10*B*, Br −4.78).

#### Regions of overlap between mPFC and amygdala labeled terminals with eyeblink-associated pontocerebellar cells

The pattern of mPFC terminals and the locations of pontine nuclei cells that project to eyeblink-associated cerebellar regions indicated two primary regions of potential interaction. The first region is in the dorsal and lateral regions of the rostral basilar pons (bregma −3.88 to −4.16; Figs. 14*B*, 11*A*). Although the population of retrogradely labeled cells was densest in the medial pons, a substantial number of cells were observed in the dorsal and lateral rostral basilar pons and overlapped with regions of dense mPFC terminal labeling, suggesting that mPFC input could directly interact with pons cells projecting to eyeblink-associated cerebellar regions. Similarly, inputs from the amygdala also overlapped with eyeblink-associated pontocerebellar cells most densely in the anterior pons, in the ventral and lateral regions (bregma −3.88 to –4.16; Figs. 14*B*, 12*B*). Furthermore, overlap between mPFC and amygdala terminal fields in the rostral-most pons represents a region of potential interaction between those inputs (Fig. 14*B*). Interestingly, the anatomical data indicate that mPFC and amygdala inputs to the rostral basilar pons would be almost exclusive to pontine cells projecting to the cerebellar cortex, because anterior deep nucleus projecting cells were only sparsely observed there (compare bregma −3.88 to −4.16; Figs. 14*B*–*D*, 11*A*,*B*, top). A second region of potential interaction between mPFC terminals and pontocerebellar neurons is the RTN, in which terminals overlapped with putative eyeblink-associated cells projecting to both the cerebellar cortex and deep nuclei (bregma −4.48 to −4.84; Figs. 14*B*,*D*, 11*A*,*B*). The data suggest that the RTN is a second potential source of mPFC input to support trace eyeblink conditioning in mice, but is an unlikely region of potential interaction with amygdala inputs given that few if any terminals were observed there (Figs. 13*D*, 14*B*,*D*).

## DISCUSSION

### Mice can learn trace conditioned responses to a light CS while head-fixed and permitted to freely run on a wheel

The first key finding of the current work is that mice can learn trace eyeblink conditioning using a visual (light) CS paired with an air puff US while head-restrained on a running wheel. Eyeblink responses were devoid of startle using these training methods, and the acquisition of CRs was dependent on a relatively restricted region of the dorsal-caudal mPFC, indicating that a trace interval as short as 250 ms is forebrain-dependent in mice. Furthermore, CRs were completely abolished by inactivation of the cerebellum, in contrast to previous work in mice in which cerebellar manipulations revealed a noncerebellar short-latency step-like component in CRs (Koekkoek et al., 2005; Boele et al., 2010; Sakamoto and Endo, 2010). Interestingly, amygdala inactivation also resulted in the near abolition of CR expression. The fact that mice showed well timed CRs, and based on known anatomical connectivity, we hypothesize that under these training conditions the amygdala is likely providing the rostral pons with an input that contributes to the CS representation that supports cerebellar learning. The data strongly suggest that mice can learn trace eyeblink conditioning in a manner that does not engage startle-associated circuitry, which allowed for more direct study of the forebrain regions that support trace eyeblink conditioning.

Because the expression of learning was not contaminated with startle-associated responses, we were able to characterize the details of the acquisition and flexible expression of learned trace eyeblink responses in mice. Previous reports using other training methods suggested that mice can learn trace conditioning very quickly, most often showing CRs in 30–60% of trials during the very first training session with performance reaching asymptote within two to five sessions (for a 250 ms trace interval; Takatsuki et al., 2001; Kishimoto et al., 2002; Tseng and O'Donnell, 2004; Weiss, 2005; Ewers et al., 2006; Galvez et al., 2009; Brown et al., 2010). Extinction training typically failed to fully extinguish responses (25–40% CR rates after 4–6 d; Takatsuki et al., 2001; Sakamoto et al., 2005). However, most of these studies also reported startle responses for at least a proportion of training trials (10–50% of trials; Tseng and O'Donnell, 2004; Ewers et al., 2006; Brown et al., 2010), leaving open the possibility that plasticity in the startle-associated circuitry may have affected learning at some point during training. Additionally, it is worth noting that fast learning, often within a single training session of a few trials, is a hallmark of amygdala-dependent fear conditioning (Milad and Quirk, 2002; Lai et al., 2012). We show here that in the absence of CS-evoked startle and freezing responses, most mice required four to eight training sessions (with baseline CR rates of 0–5%) before beginning to express CRs, which gradually increased over an additional approximately five sessions. Once learning began, the progression of learning was similar across mice and independent of the number of sessions needed before learning was observed, similar to the learning curves often reported for rabbits (Kalmbach et al., 2010b; Siegel, 2014). Furthermore, using the current training procedures mice were able to fully extinguish CRs within two to three training sessions (0–5% CR rates) and reacquired CRs to previous levels of performance within five sessions. Previous studies might suggest that training stimuli that induce startle responses appear to accelerate aspects of the learning and perhaps result in robust responses that are resistant to extinction. However, establishing which forebrain regions are necessary for trace eyeblink conditioning and attempting to determine their precise roles would be intractable under such conditions, given the interactions and/or parallel functions of some these brain regions in mediating startle responses (Koch, 1999; Rosen and Davis, 1988).

Similar to recent reports for delay conditioning (Chettih et al., 2011; Heiney et al., 2014), we observed timing in the latency to the peak amplitude of CRs in mice, while the onset of learned responses was similar for different training intervals. Therefore, a notable species-specific difference between mice and rabbits is that rabbits show timing to different training intervals as a shift in the latency to onset of CRs, with the overall topography of the CR appearing similar for different training intervals (Boneau, 1958; Ohyama et al., 2003, 2010). The data presented here suggest that mice time their CRs relative to the training interval used by adjusting the initial velocity of CRs (Chettih et al., 2011), whereas rabbits time learned responses by waiting the appropriate amount of time before initiating a response (Ohyama et al., 2003). Both of these strategies result in eyelid closures that are adaptive and anticipate the timing of the US, although the cerebellar mechanisms modulating each are likely different (Medina et al., 2000; Chettih et al., 2011). The timing of CRs in mice was appropriate to the interstimulus interval at the earliest observable points during learning, and was insensitive to breaks in training (7 or 14 d) and extinction or reacquisition training. The amplitude of CRs was more sensitive, however, and was significantly affected by long breaks in training. In fact, modulation of CR amplitude appeared to mediate the expression of responses during extinction and reacquisition. Little is known about how the cerebellum specifically modulates the amplitude of motor responses, and probably represents one of the least studied aspects of the behavior (but see Kreider and Mauk, 2010). However, the current data and a recent report (Heiney et al., 2014) suggest that increases in CR amplitude represents a slow process dissociable from the initial learning because increases are observed throughout acquisition and continue to be observed for many additional sessions while performance is asymptotic, until full amplitude responses are achieved.

### Trace eyeblink conditioning depends on a specific region of the mPFC in mice, the output of which overlaps with pontine cells that project to critical eyeblink-associated regions in the anterior cerebellum

A critical finding of the current work was that acquisition of cerebellar-dependent trace CRs was blocked by relatively localized lesions of the dorsal mPFC (∼500 µm along the anterior–posterior axis). This same mPFC region was also shown to support the postacquisition expression of CRs. To our knowledge, this is the first demonstration of mPFC-dependent trace conditioning in a mouse model and further supports previous data indicating that an interval as short as 250 ms is indeed forebrain-dependent in mice (Tseng et al., 2004). The data presented here also suggest that trace intervals approaching 500 ms appear to be beyond that which mice can learn or show well timed responses (Tseng et al., 2004). Although we used a CS length that is a fair amount shorter than most previous studies used (50 vs 250 ms; Tseng et al., 2004), our lesion results suggest that for trace conditioning, it is the length of the stimulus-free trace interval that determines forebrain involvement, and not the length of the interstimulus interval per se (Kalmbach et al., 2010b).

Previous lesion work in other species typically used fairly large mPFC lesions, often comparing animals with rostral or caudal mPFC damage, and so investigators were typically unable to specify whether a more specific critical region existed within those areas (Kronforst-Collins and Disterhoft, 1998; Takehara et al., 2003). Infusion studies suggested that restricted disruptions of the mPFC are sufficient to cause a deficit in performance after learning (Kalmbach et al., 2009; Chen et al., 2014), but are of limited use in determining a precise functional location given that pharmacological manipulations can vary from session to session and can fail for a given test session for a variety of reasons, even if infusion cannula are well placed. Therefore, a null result from an infusion test cannot provide much information regarding locations that do not support a task. Our use of relatively restricted lesions, and the clear discrimination between learners and nonlearners, allowed us to localize a region within the mPFC critical for the acquisition of trace eyeblink CRs. Specifically, the data indicate that disruption of the AGm/ACc over as little as 500 μm along the anterior–posterior axis between bregma +0.75–1.75 is sufficient to block learning in mice. Because a relatively precise mPFC region was first identified by the lesion study, we were then able to more reliably test the importance of this same region for postacquisition expression using unilateral infusions of muscimol in trained animals. The importance of this region of mPFC for trace eyeblink conditioning is in good agreement with the results obtained from other species (Kronforst-Collins and Disterhoft, 1998; Weible et al., 2003; Kalmbach et al., 2009; Siegel et al., 2012; Chen et al., 2014; but see Mclaughlin et al., 2002). Note that damage to the prelimbic region was not necessary to block acquisition in mice in the current study, and that lesions caudal to bregma +0.75 resulted in learned responses with distinctly abnormal topographies that are not easily explained.

Previous work has investigated prefrontopontine and pontocerebellar projection patterns in several species under various contexts, including rodents and rabbits with regard to eyeblink conditioning (Buchanan et al., 1994; Weible et al., 2007; Moya et al., 2014). However, we were able to focus specifically on projections from the mPFC region that our lesion experiment indicated was necessary for the acquisition and expression of CRs. The same animals provided a functional basis for the location of retrograde tracer infusions to label pontine cells from eyeblink-associated cerebellar regions. We show here that the mPFC of mice projects most densely to the anterior basilar pons, as observed in rats (Wiesendanger and Wiesendanger, 1982; Leergaard and Bjaalie, 2007; Moya et al., 2014). In contrast to rats, this projection was typically unilateral in most mice (Fig. 14*B*,*D*, compare summary data in magenta shading to Moya et al., 2014). The efficacy of our unilateral mPFC muscimol infusions on the expression of CRs is consistent with the anatomical findings (Fig. 7), suggesting that the sparse contralateral inputs observed in some of the mice do not appear to support the task. Relatively dense and expansive mPFC terminal fields were also observed in the reticulotegmental nucleus of the pons in the current study. Retrograde label from the cerebellar cortex revealed pontocerebellar cells along the entire anterior–posterior axis of the basilar and reticulotegmental nuclei of the pons, including a substantial subset that overlapped with mPFC labeled terminals. Infusions of tracer into the cerebellar anterior deep nuclei labeled somata in the RTN, a proportion of which also overlapped with mPFC terminals. However, only a sparse proportion of pontonuclear cells overlapped with mPFC terminal fields in the basilar pons. The data suggest that prefrontal cells are in an anatomical position to directly influence cerebellar-projecting neurons that could support eyeblink conditioning. One caveat to this interpretation is that non-eyeblink-associated cerebellar cells in the vicinity of the infusion sites would also take up the tracer, and so the retrogradely labeled somata would obviously also include cells that may not support eyeblink conditioning. Additionally, the strongest somatic labeling would result from uptake of retrograde tracer by pontocerebellar terminals nearest to the injection site, where the concentration of dextran was the highest. Therefore, the well labeled somata identified by our procedures were likely to project to a more restricted region of cerebellar cortex or deep nuclei than was affected by our muscimol infusions.

The central nucleus of the amygdala also projects to the rostral basilar pons, as previously reported (Mihailoff et al., 1989), and overlapped with regions in which pontocerebellar eyeblink-associated neurons were observed. Few if any terminal fields were observed in the RTN, however, suggesting that if amygdala inputs do indeed facilitate pontine responses to other CS-associated inputs, such as from the mPFC, this must occur in the rostral-most basilar pons. Alternatively, it is possible that the amygdala provides a parallel additional input that contributes to the CS representation. In either potential scenario, if the CS representation is perturbed during postlearning training sessions (for example, by blocking a previous source of CS-associated input), the pattern of inputs to the cerebellum would be altered and fail to generate a CR. It is unlikely that central amygdala inactivations could have affected the critical region of mPFC directly, because amygdala terminals were not observed there. Furthermore, it is also unlikely that any feedback from the central nucleus to other amygdala nuclei, which do project to the mPFC, could have had an indirect effect because the central nuclei show extremely sparse (if any) feedback to other amygdaloid nuclei (Jolkkonen and Pitkänen, 1998). It should be noted, however, that our infusions systematically passed through the caudolateral globus pallidus in targeting the central nucleus, and so it is possible that this region was also affected by both the muscimol and anterograde tracer infusions. However, we were unable to find any studies describing pontine inputs from the globus pallidus, and so the anatomical findings reported here are likely the results of central nucleus uptake. It is more difficult to rule out whether the observed performance deficits might be entirely or partially the result of globus pallidus inactivation, but given the apparent lack of an anatomical pathway to support a role for this region in trace eyeblink conditioning it seems unlikely. Although we used bilateral infusions of muscimol in the current study, the strictly unilateral nature of amygdalopontine inputs indicates that unilateral manipulations of the amygdala should yield equivalent results.

#### Summary

In the current work, we have established and characterized trace eyeblink conditioning in a mouse model devoid of CS-evoked startle responses. Under these conditions, the associative learning depends on a restricted region of the mPFC, and the cerebellum drives the CRs with no extra-cerebellar motor component as previously observed in mice. In the process of identifying the brain circuitry that supports trace eyeblink conditioning in mice, we revealed a more precise role for the central nucleus of the amygdala in this task as providing a CS-associated input to the cerebellum (via the rostral basilar pons) that appears to contribute to the overall CS representation. Additional studies are required to determine whether this role is necessary for acquisition (as shown here for the mPFC) or facilitatory, as previously suggested (such as potentiating pontine inputs from other brain regions; Boele et al., 2010). Nevertheless, the methods and data reported here provide an essential substrate for future experiments focused on understanding how identified brain regions support this basic form of associative learning and memory, including its modulation and expression.

## Synthesis

The decision was a result of the Reviewing Editor Christian Hansel and the peer reviewers coming together and discussing their recommendations until a consensus was reached. A fact-based synthesis statement explaining their decision and outlining what is needed to prepare a revision is listed below. The following reviewers agreed to reveal their identity: John Disterhoft

We have now received comments on the revised manuscript from the two expert reviewers that have also seen the first version of the paper. Both referees feel that the manuscript has been significantly improved and all major concerns have been addressed. There are several minor issues that were raised that need to be addressed before moving to the next stage. Please submit a revised manuscript as early as possible. The new version will not be sent back to the original reviewers, but will be looked at by the editor only.

This manuscript is much improved over the previous version. The authors have discovered that their trace eyeblink conditioning task in mice is dependent not only on the medial prefrontal cortex but also on the central nucleus of the amygdala. And they have determined parameters that allow trace eyeblink conditioning to be done with the presence of no or very few startle responses. This makes the task in mice much more comparable to the same task previously studied in the rabbit and rat.

1. The author(s) responded very thoroughly to the suggestions made regarding the first submission. Unfortunately the new version is rather encyclopedic and includes several elements that have been previously published. The rotating cylinder was described by the Medina laboratory, the involvement of the amygdala in conditioning (although not trace eyeblink conditioning) has a rich history, especially by Michael Gabriel (who is not cited, e.g. PMID: 15506877), and a visual CS (white light) was used to eyeblink condition mice with little startle activity by Weiss et al. (2002, PMID: 12586551). The new results appear to be the identification in mice of the critical region in the cerebellum, frontal cortex and amygdala and their joint projection to the pontine nuclei (which are similar to what has been described in rabbits). However, the previously studied elements are brought together in a way that will be useful to others in the field.

2. The authors used a 50 ms CS and a trace interval of 250 ms. Most other trace conditioning studies use a 250-500ms CS. A potential confound here is that the interstimulus interval (ISI) used with a 50ms CS creates a 300ms ISI (as opposed to a 500-750ms ISI) which is rather close to the 250ms ISI that appears to be ideal for delay conditioning, and thought to be mediated by LTD of parallel fiber-Purkinje cell synapses. This could account for well-timed CRs without the need for any forebrain input, although the lesion results are compelling and argue against this.

3. The authors performed the amygdala inactivations in response to suggestions of the previous review. The results are quite interesting and add an interesting complexity to the story. Although the authors clearly are interested in emphasizing the medial prefrontal cortex involvement in trace eyeblink conditioning, they shouldn't back away from their interesting amygdala inactivation results. Specifically, on lines 1099-1100, it is stated that further studies are required to show that the amygdala is required for trace eyeblink conditioning. The data shown here support that amygdala is required for performance of this learned task.

Other comments:

L. 199, the sagittal sinus (a vein) is referred to, not an artery.

L. 276, Most pseudoconditioning is designed to maintain the same stimulus density over the course of a training session. Here the mice were given the US on every fifth trial which would probably minimize any pseudoconditioning that may have occurred if the stimulus density was maintained.

L. 403-408, the author(s) list the different statistical tests done, depending on sample size, and use an unusual method of "foot-noting" many analyses that refers the reader to the table where power calculations are listed. The results would seem more reliable if the Ns were increased rather than trying to explain the impact of results that are underpowered.

L. 420, trials in which the wheel was being moved were analyzed, but the percentage of those trials was not stated.

Figure 1- data are from which session? What intensity of blue light was used, or the part number and the current (mA) applied. Fig 1D- does the purple color indicate no movement- the color scale indicates no movement as dark blue. Fig 1E- the smaller amplitudes at 1500 fps may be due to the camera being less sensitive to light at the faster frame rate.

Figure 7- Should comment on how a unilateral lesion would affect behavior. Most lesions of hippocampal/prefrontal sites require bilateral lesions to get effects.

The authors did an excellent job in revising the manuscript. The remark about the paper by Boele et al., 2010 was not meant to be removed, but to also add the original work, which is now added. Please keep the ref to Boele et al. (2010) in the text at the appropriate places and ref list, as it provides relevant concepts and data to this manuscript. Otherwise fine.

Please find our responses to the reviewers' comments below:

This manuscript is much improved over the previous version. The authors have discovered that their trace eyeblink conditioning task in mice is dependent not only on the medial prefrontal cortex but also on the central nucleus of the amygdala. And they have determined parameters that allow trace eyeblink conditioning to be done with the presence of no or very few startle responses.

This makes the task in mice much more comparable to the same task previously studied in the rabbit and rat.

1. The author(s) responded very thoroughly to the suggestions made regarding the first submission. Unfortunately the new version is rather encyclopedic and includes several elements that have been previously published. The rotating cylinder was described by the Medina laboratory, the involvement of the amygdala in conditioning (although not trace eyeblink conditioning) has a rich history, especially by Michael Gabriel (who is not cited, e.g. PMID: 15506877), and a visual CS (white light) was used to eyeblink condition mice with little startle activity by Weiss et al. (2002, PMID: 12586551). The new results appear to be the identification in mice of the critical region in the cerebellum, frontal cortex and amygdala and their joint projection to the pontine nuclei (which are similar to what has been described in rabbits). However, the previously studied elements are brought together in a way that will be useful to others in the field.

We fully acknowledge the fact that Medina and colleagues developed important innovations for eyeblink training in mice, including use of a rotating cylinder (Introduction, p. 6, Methods, p. 10). We also acknowledge previous work indicating the involvement of the amygdala in delay eyeblink conditioning and theories associated with those findings (Introduction, p. 5). The use of a light CS by Weiss et al., 2002 was not cited because the authors made no reference to why they used a light, nor did they measure or report anything regarding startle responses. The single trial example they showed did not indicate a startle response on that one trial, but overall their window of CR analysis did not include the first 50 ms of the trial, so the presence or absence of startle cannot be inferred from the data presented in that study. Second, a literature search of Michael Gabriel revealed that his work on the amygdala focused on an instrumental avoidance conditioning task and not eyeblink conditioning. In fact, a quote from a recently authored book chapter suggests that he never demonstrated a connection between the amygdala and eyeblink conditioning: "A partial dissociation was also found with respect to the amygdala, insofar as the amygdala is essential for discriminative avoidance learning but not for NM conditioning." (p. 152, Gabriel and Talk, "A Tale of Two Paradigms: Lessons Learned From Parallel Studies of Discriminative Instrumental Learning and Classical Eyeblink Conditioning" in Model Systems and the Neurobiology of Associative Learning, Psychology Press, 2013.)

Therefore, we feel that discussion with regard to these issues was handled appropriately and we stand by the citations given as appropriate and inclusive.

2. The authors used a 50 ms CS and a trace interval of 250 ms. Most other trace conditioning studies use a 250-500ms CS. A potential confound here is that the interstimulus interval (ISI) used with a 50ms CS creates a 300ms ISI (as opposed to a 500-750ms ISI) which is rather close to the 250ms ISI that appears to be ideal for delay conditioning, and thought to be mediated by LTD of parallel fiber-Purkinje cell synapses. This could account for well-timed CRs without the need for any forebrain input, although the lesion results are compelling and argue against this.

We agree. Perhaps, for trace conditioning, it is important to consider not only the ISI, but more importantly the length of the stimulus-free trace interval as key to forebrain involvement. We have added a statement pointing this out in the Discussion (p. 44).

3. The authors performed the amygdala inactivations in response to suggestions of the previous review. The results are quite interesting and add an interesting complexity to the story. Although the authors clearly are interested in emphasizing the medial prefrontal cortex involvement in trace eyeblink conditioning, they shouldn't back away from their interesting amygdala inactivation results. Specifically, on lines 1099-1100, it is stated that further studies are required to show that the amygdala is required for trace eyeblink conditioning. The data shown here support that amygdala is required for performance of this learned task.

Our intention in making this statement was to acknowledge a theory proposed by others in the literature with regards to delay eyeblink conditioning (specifically, that the amygdala facilitates other pontine inputs), which our data does not specifically address. We have reworded this statement to avoid the misperception (Discussion, p. 49).

Other comments:

L. 199, the sagittal sinus (a vein) is referred to, not an artery. - This has been corrected (Methods, p. 8). L. 276, Most pseudoconditioning is designed to maintain the same stimulus density over the course of a training session. Here the mice were given the US on every fifth trial which would probably minimize any pseudoconditioning that may have occurred if the stimulus density was maintained.

The overall concern was to test whether mice developed eyelid closures due to sensitization to the light with repeated exposure. To address this it was important to keep the density of light presentations similar to a typical training session. We now explicitly state this in the Methods (p. 11). L. 403-408, the author(s) list the different statistical tests done, depending on sample size, and use an unusual method of "foot-noting" many analyses that refers the reader to the table where power calculations are listed. The results would seem more reliable if the Ns were increased rather than trying to explain the impact of results that are underpowered.

The issue of Ns was addressed in the previous response to reviewers, and acknowledged in the Methods (p. 17). In the end, the ONLY experiment for which this discussion applies is for mice trained with the 50-450 protocol because few mice were able to learn this interval. The raw data clearly argues against a false negative in this case (Fig. 3B). The style of annotation and the power table were done in accordance with the journal's specifications. L. 420, trials in which the wheel was being moved were analyzed, but the percentage of those trials was not stated.

This is now stated in the Methods (p. 18).

Figure 1- data are from which session?

-The figure legend now specifies this.

What intensity of blue light was used, or the part number and the current (mA) applied.

-This is now given in the Methods (p. 10).

Fig 1D- does the purple color indicate no movement- the color scale indicates no movement as dark blue.

-There actually was a strip of purple at the bottom of the color scale, which was difficult to detect given its size. We have increased the purple so that it is easier to see in the color scale (Fig. 1D). Fig 1E- the smaller amplitudes at 1500 fps may be due to the camera being less sensitive to light at the faster frame rate.

-The amplitudes observed at 1500 fps did not appear smaller to our eyes.

In fact, a range of large and small startle responses can be seen in both 200 fps and 1500 fps examples. In any event we compensated for the darker image by appropriately adjusting the threshold, using the airpuff response as a reference.

As observed in the figure, the URs are comparable between the two sampling rates.

Figure 7- Should comment on how a unilateral lesion would affect behavior. Most lesions of hippocampal/prefrontal sites require bilateral lesions to get effects.

-We have added comments regarding our predictions in the Discussion (p. 45, 46, 49).

The authors did an excellent job in revising the manuscript. The remark about the paper by Boele et al., 2010 was not meant to be removed, but to also add the original work, which is now added. Please keep the ref to Boele et al. (2010) in the text at the appropriate places and ref list, as it provides relevant concepts and data to this manuscript. Otherwise fine.

-We have included this citation where appropriate (Introduction, p. 5; Discussion, pp. 41, 49).
